# Elucidating the pharmacodynamic mechanisms of Yuquan pill in T2DM rats through comprehensive multi-omics analyses

**DOI:** 10.3389/fphar.2023.1282077

**Published:** 2023-11-17

**Authors:** Yan Lei, Jianmei Huang, Zhongshui Xie, Can Wang, Yihong Li, Yutong Hua, Chuanxin Liu, Ruijuan Yuan

**Affiliations:** ^1^ School of Chinese Materia Medica, Beijing University of Chineses Medicine, Beijing, China; ^2^ Medical Key Laboratory of Hereditary Rare Diseases of Henan, Department of Metabolism and Endocrinology, Endocrine and Metabolic Disease Center, The First Affiliated Hospital, College of Clinical Medicine of Henan University of Science and Technology, Luoyang Sub-center of National Clinical Research Center for Metabolic Diseases, Luoyang, China

**Keywords:** Yuquan pill, type 2 diabetes, serum pharmacochemistry, network analysis, transcriptomics

## Abstract

**Background:** Yuquan Pill (YQW) is a modern concentrated pill preparation of six herbs, namely, Ge Gen (*Pueraria lobata* Ohwi), Di huang (*Rehmannia glutinosa* Libosch.), Tian Huafen (*Trichosanthes kirilowii* Maxim.), Mai Dong (*Ophiopogon japonicus* (L. f.) Ker Gawl.), Wu Weizi (*Schisandra chinensis* (Turcz.) Baill.) and Gan Cao (*Glycyrrhiza uralensis* Fisch.). It is extensively used to treat type 2 diabetes-related glucose and lipid metabolism disorders. But what’s the pharmacodynamic substance and how it works in the treatment of Type 2 diabetes mellitus (T2DM) are still unclear.

**Purpose:** The purpose of this study is to determine the likely pharmacological components and molecular mechanism of YQW’s intervention on T2DM by combining serum pharmacochemistry, network analysis and transcriptomics.

**Methods:** The efficacy and prototypical components of blood entry were determined after oral administration of YQW aqueous solution to T2DM rats induced by high-fat feed and low-dose streptozotocin (STZ), and the key targets and pathways for these compounds to intervene in T2DM rats were predicted and integrated using network analysis and transcriptomics techniques.

**Results:** In diabetic rats, YQW can lower TG, CHO, NO, and MDA levels (*p* < 0.05) while increasing HDL-C levels (*p* < 0.01), and protecting the liver and kidney. 22 prototype components (including puerarin, daidzein, 3′-methoxypuerarin, and liquiritigenin, among others) were found in the serum of rats after oral administration of YQW for 90 min, which might be used as a possible important ingredient for YQW to intervene in T2DM rats. 538 YQW pharmacodynamic components-related targets and 1,667 disease-related targets were projected through the PharmMapper database, with 217 common targets between the two, all of which were engaged in regulating PI3K-Akt, MAPK, Ras and FoxO signal pathway. Finally, the mRNA expression profiles of liver tissues from rats in the control, model, and YQW groups were investigated using high-throughput mRNA sequencing technology. YQW can regulate the abnormal expression of 89 differential genes in a disease state, including 28 genes with abnormally high expression and 61 genes with abnormally low expression. Five common genes (Kit, Ppard, Ppara, Fabp4, and Tymp) and two extensively used regulatory pathways (PI3K-Akt and MAPK signaling pathways) were revealed by the integrated transcriptomics and network analysis study.

**Conclusion:** The mechanism of YQW’s intervention in T2DM rats could be linked to 22 important components like puerarin, daidzein, and glycyrrhetinic acid further activating PI3K-Akt and MAPK signaling pathways by regulating key targets Kit, Ppard, Ppara, Fabp4, and Tymp, and thus improving lipid metabolism disorder, oxidative stress, and inflammation levels in T2DM rats. On the topic, more research into the pharmacological ingredient foundation and mechanism of YQW intervention in T2DM rats can be done.

## 1 Introduction

About 425 million people in the world suffer from diabetes, of which T2DM and its complications affect the great majority of them (Guariguata et al., 2014; Thomas, 2022). According to statistics published by the International Diabetes Federation, by 2045, there will be around 629 million individuals worldwide with diabetes, of which T2DM patients account for more than 90% of the total (Forouhi et al., 2018; Kapoor et al., 2021). T2DM is a chronic disease impacted by the environment, genetics, and multiple genes. T2DM and its severe effects, such as kidney damage, nerve damage, retinopathy, and cardiovascular disease, are a serious hazard to human health worldwide (Bhatti et al., 2016; Kanter and Bornfeldt, 2016). Diabetes currently has no remedy anywhere in the planet. Only hypoglycemic drugs, a nutritious diet, regular exercise, self-monitoring blood sugar dynamics, and psychological modifications can help diabetics alleviate their symptoms and prevent difficulties (Kirchner et al., 2013; Ning et al., 2022). Traditional Chinese Medicine (TCM) has a long history of treating diabetes (Zheng et al., 2021). According to TCM, the main causes of DM are yin-jin deficiency, as well as excessive dryness and heat. Patients with a qi and yin deficiency, dryness and heat, vein and tendon obstruction, and tendons and vein dystrophy are common in TCM clinical diagnosis and therapy, causing injury to the viscera and organs. To improve the overall condition of T2DM patients and achieve a better curative effect, TCM techniques such as tonifying qi, nourishing yin, and clearing away heat should be used to exert the effects of nourishing yin, promoting body fluid production, quenching thirst, relieving vexation, invigorating qi, and harmonizing the middle warmer (Chen et al., 2016; Wong, 2016).

YQW is a modern polyherbal formulation modified from Yuquan Powder in Volume II (Diabetes) of the ancient book Zhongfutang Public Selection Recipe, which made up of six medications: *Pueraria lobata* (Willd.) Ohwi (gegen), *Rehmannia glutinosa* (Gaertn.) DC. (dihuang), *Ophiopogon japonicus* (Thunb.) Ker Gawl. (maidong), *Trichosanthes kirilowii* Maxim./*Trichosanthes rosthornii* Harms (tian huafen), *Schisandra chinensis* (Turcz.) Baill. (wu weizi) and *Glycyrrhiza uralensis* Fisch./*Glycyrrhiza inflata* Batalin/*Glycyrrhiza glabra* L. (gancao). Gegen is a monarch drug that enhances fluid production, quenches thirst, and reduces fever. It belongs to the lung and stomach meridians (Chen et al., 2016). Ministerial remedies dihuang nourishing yin; tian huafen, thirst-quenching, heat-clearing and fire-purging; wu weizi nourishes qi, produces body fluid, and astringes astringent, both of which are complementary drugs (Tan et al., 2017). Gancao clears heat and toxic materials, tonifies spleen, invigorate qi, and is used as an agent in YQW, which can be used to harmonize various drugs (Liu et al., 2021). YQW has been shown to treat symptoms of T2DM patients in clinical trials. Gegen has pharmacological effects of lowering blood sugar and regulating blood lipid (Liu et al., 2022b); dihuang can lower blood sugar and diuresis (Li et al., 2022a; Yang et al., 2022b); maidong can not only lower blood sugar (Li et al., 2022a), but also has many important pharmacological effects such as anti-myocardial ischemia. The majority of current studies, however, are focused on YQW’s therapeutic efficacy in the treatment of T2DM. There has been no conclusive research or conclusion as to what the material base of its complete prescription is or how to use it to treat T2DM.

Therefore, this subject systematically analyzed the potential pharmacodynamic substances and mechanism of YQW intervention in T2DM rats using the serum pharmacochemistry research method, combining with network analysis and transcriptomics technology.

## 2 Materials and methods

### 2.1 Reagents and materials

Yuquan Pill (batch No. 200401, Chengdu Jiuzitang Jinding Pharmaceutical Co., Ltd., China); Metformin Hydrochloride Tablets (batch No. H20023370, Bristol-Myers Squibb., China); Streptozotocin (batch No. 18883-66-4, Shanghai yuanye Bio-Technology Co., Ltd., China); Uralose (CAS No. #51-79-6, Shanghai yuanye Bio-Technology Co., Ltd., China); Citric Acid-Sodium Citrate Buffer (batch No. L10S11G124133, Shanghai yuanye Bio-Technology Co., Ltd., China); 45% High-fat feed (batch No. D12451. SPF (Beijing) Biotechnology Co., Ltd., China); NO assay kit (batch No. E1030. Beijing Pulley Gene Technology Co., Ltd., China); MDA test kit (batch No. 20201106. Nanjing Jiancheng Bioengineering Institute, China); mass spectrometry grade methanol, acetonitrile and formic acid (Thermo Fisher Company, United States); ultrapure water (A.S.WATSON TM LIMITED); ME155DU electronic balance (METTLER TOLEDO, China); JA 2003B electronic balance (Shanghai Yueping Scientific Instruments Co., Ltd., China); KQ-300DB ultrasonic cleaning machine (Kun Shan Ultrasonic Instruments Co., Ltd., China); high-speed desktop refrigeration centrifuge (Shanghai Anting Scientific Instrument Factory, China); Pipette (Eppendorf, Germany); Fully automated biochemistry analyser (BECKMAN COULTER); EPOCH enzyme labeller (BioTek); Eclipse Ti-E laser confocal microscope (NIKON, Japan); Panoramic section scanner, 3DHISTECH (HUNGARY); ACQUITY I-CLASS UPLC (Waters, United States), SYNAPT G2-Si Q-TOF MS (Waters, United States).

### 2.2 YQW aqueous/alcohol solution preparation

2.0 g of YQW (Concentrated Pill) powder were weighed and ultrasonic extracted for 20 min with 20 mL of 75% methanol solution (240 W, 25 kHz). The mixture was centrifuged twice at 13,000 r/min for 15 min each time after cooling to room temperature and the supernatant was removed.

### 2.3 Animal experiments

#### 2.3.1 Experimental animals

SPF grade SD rats (male, 4 weeks old, 100 ± 10 g) were acquired from SPF (Beijing) Biotechnology Co., Ltd. (Production license number: scxk (Beijing) 2019-0010, Quality certificate of experimental animals: No. 1103242011017624). These rats were cared and treated according to the regulations and general recommendations of China Laboratory Animal Management Regulations, and approved by the Animal Ethics Committee of Beijing University of Traditional Chinese Medicine (No. BUCM-4-2020081003-3147; Date: 10-08-2020).

#### 2.3.2 Animal grouping and modeling

After 1 week of adaptive feeding in a standard environment, 10 rats were randomly selected as the control group and the remaining rats were fed 45% high-fat feed as the modeling group using the random number approach. The modeling rats received a single intraperitoneal injection of STZ 35 mg/kg (Jawale et al., 2016) after 5 weeks, while the control rats received an identical volume of citric acid-sodium citrate buffer (0.01 mol/L, pH 4.5). A random blood glucose measurement of 16.7 mmol/L was regarded a successful modeling 72 h after injection (Yang et al., 2022c). Then, based on their blood glucose levels, we separated successfully modeled rats into four groups: Model group, Metformin (Met) group, YQW high-dose (YQW-H) group, and YQW low-dose (YQW-L) group. On the basis of the model group, varied concentrations of YQW (YQW-H group: 3.95 g/kg; YQW-L group: 0.99 g/kg) and positive medication (Met group: 0.34 g/kg) were administered to the administration groups. The rats’ activity, body weight, and physiological conditional were all monitored during the intervention.

#### 2.3.3 The effect of YQW on serum biochemical indexes and organ indexes

Finally, all rats were sacrificed using anesthetics following a 12-h fast, in accordance with laboratory animal ethics. To get the serum, a blood sample was taken from the abdominal aorta and centrifuged at 3,000 r/min for 15 min at 4°C. The supernatant was taken out of the mixture and split. A completely automated biochemical analyzer was used to assess blood glucose, TG, CHO, HDL-C, LDL-C, and SOD. NO and MDA were also measured according to the kit’s specifications, and the remaining serum was kept at −80°C until needed. The splenic glands were removed and washed with saline after the rats were executed, and the blood was removed from the surface with filter paper before being weighed on an electronic balance and the splenic index calculated.

#### 2.3.4 Histopathological observation

The liver and kidney were embedded in paraffin, dehydrated, and stained with H&E, and examined under a light microscope at 20 and 200 magnification after being fixed in 4% paraformaldehyde for 48 h.

#### 2.3.5 Data processing

The data is presented as continuous variables or qualitative descriptions depending on the type of data. GraphPad Prism 5 software was used to process the continuous variables, and the results were reported as mean ± SD. A statistically significant difference was defined as *p* < 0.05.

### 2.4 Pharmaceutical chemistry research on serum

#### 2.4.1 Method of administration to rats

Blood was taken from the inner orbital canthus of the rats in the dosing group at 45 min after oral administration in the morning; at 90 min, the rats in each group were anesthetized with 25% urethane (*m/V*) by intraperitoneal injection (dose administered was 0.4 mL/100 g) and blood was taken from the abdominal aorta.

#### 2.4.2 Blood samples pretreatment

To obtain the drug-contained serum, the whole blood was centrifuged at 3,000 r/min (4°C, 15 min) after standing for 30 min. The sera of rats in each group were pooled at 45 and 90 min, then 1,000 μL of the combined serum were added 3 times of acetonitrile, vortex for 2 min, centrifuged at 3,000 r/min (4°C, 15 min), and then blew dry with nitrogen at 40°C and redissolved with 100 μL methanol (enrichment 10 times). Finally, the redissolution fluid was centrifuged at 12,000 r/min (4°C, 15 min), and the supernatant was separated for sample injection analysis using UPLC-Q-TOF/MS.

#### 2.4.3 Chromatographic and mass spectrometric conditions

Chromatographic column: Waters ACQUITY BEH C18 column (2.1 mm × 100 mm, 1.7 μm); flow rate: 0.3 mL/min; column temperature: 35°C; injection volume: 5.0 μL.mobile phase: 0.1% formic acid in water (A) - acetonitrile (B); gradient elution procedure: (1) condition 1: 0–2 min, 5% B ∼ 5% B; 2–17 min, 5% B ∼ 98% B; 17–20 min, 98% B ∼ 98% B; 20–23 min, 98% B ∼ 5% B; 23–25 min, 5% B ∼ 5% B; (2) condition 2: 0–8 min, 2% B ∼ 9% B; 8–18 min, 9% B ∼ 32% B; 18–20 min, 32% B ∼ 89% B; 20–35 min, 89% B ∼ 100% B; 35–37 min, 100% B ∼ 2%B; 37–40 min, 2% B ∼ 2% B (Note: condition 1 was used to collect the chromatogram of the YQW solution, and condition 2 was used for the collection of the rat *in vivo* blank and drug-contained serum).

In both positive and negative ionization modes, an electrospray ionization source (ESI) was used for mass spectrometry analysis. Leucine-enkephalin was employed as an external reference for real-time calibration to guarantee that correct mass numbers were collected for each component of the molecule during data capture, yielding fragment ions *m/z* 556.2721 [M+H]^+^. As an auxiliary spray ionization and desolventizing gas, high purity N_2_ was utilized. The drying gas flow rate was 10 mL/min, the N_2_ temperature was 120°C, the nebulisation chamber air pressure was 310 kPa, the desolventizing nitrogen flow rate was 900 L/h, the cone hole backblast nitrogen flow rate was 50 L/h, the capillary ionisation voltage was 500 V, the cone hole voltage was 40 V, the collision energy was 40–65 eV, and the data acquisition range was *m/z* 50-1200Da.

#### 2.4.4 Establishment of the YQW database

To collect information on the chemical composition of six herbs in YQW, the following online databases were used: TCMID (http://www.megabionet.org/tcmid), TCMSP (http://tcmspw.com/tcmsp.php), ETCM (http://www.ehbio.com/ETCM), BATMAN-TCM (http://bionet.ncpsb.org/batman-tcm/). By means of TCM database, the exclusive database of YQW was created, and information on the chemical composition of the six herbs was collected, including chemical names, molecular formulas, structural formulae, and molecular weights.

#### 2.4.5 Identification of chemical composition

Masslynx V4.1 (Waters, United States) and UNIFI were utilized to process data and the unique database of YQW were used to identify chemical compositions. The major chemical composition of YQW was then identified and confirmed by extracting fragment ions and comparing it to relevant literature data based on the retention time (RT) and mass spectrum information of each chemical component under positive and negative ion modes.

#### 2.4.6 Acquisition of the target

The PharmMapper database (http://www.lilab-ecust.cn/pharmmapper/) was employed to anticipate the appropriate chemical component-targets in the prescription given above. Then, the GeneMANIA (http://genemania.org) protein interaction analysis tool was used to predicte the indirect targets connected to the direct targets. Finally, the Uniport (http://www.uniprot.org/) database was used to convert all targets into conventional gene names.

The term “type 2 diabetes” was used to found the targets associated to T2DM by searching the Comparative Toxicogenomics Database (http://ctdbase.org/), Therapeutic Target Database (http://db.idrblab.net/ttd/), and GeneCards (http://www.genecards.org/) illness databases. The CTD database was filtered by looking for reported targets associated with T2DM that were tagged with “marker/mechanism”; the TTD database was filtered by looking for databases that clearly contained the disease “type 2 diabetes”; and the GeneCards database was filtered by having a relevance score of 26 or higher. Then, in Uniport, T2DM-targets were changed into the standard gene name. The targets of YQW in the treatment of T2DM were determined by intersecting the T2DM and composition targets.

#### 2.4.7 Protein-protein interactions (PPI) analysis

To obtain the PPI network, the YQW targets in the therapy of T2DM were submitted to the String (https://cn.string-db.org) analysis platform. The network topology of the PPI network was then analyzed using Cytoscape 4.6.1 (National Institute of General Medical Sciences, United States). According to the Degree value, the top five targets were chosen as critical objectives.

#### 2.4.8 GO and KEGG enrichment analysis

All of the above-mentioned targets were uploaded to the String analysis platform’s “Multiple Proteins” section, and “*Rattus norvegicus*” was chosen for Gene Ontology (GO) and Kyoto Encyclopedia of Genes and Genomes (KEGG) studies. The top 10 pathways in KEGG Pathways with different classification functions were chosen based on their *p* values and imported into the Omicshare (https://www.omicshare.com/) platform for pathway enrichment analysis to create a bubble map of signaling pathways; at the same time, the top 10 pathways in BP, CC, and MF were chosen based on their *p* values and imported into the microbiology online database to investigate the possible mechanism of YQW.

#### 2.4.9 Construction of the “TCM-component-target” network

Using the merge integration function, the “TCM-Component-Target” network was built in Cytoscape 4.6.1.

### 2.5 Transcriptomic studies

#### 2.5.1 RNA extraction, quality control and RNA sequencing

Shanghai Personal Biotechnology Co., Ltd. (http://www.personalbio.cn/) extracted and analysed nine samples from the three groups of rats. Electrophoresis, gel imaging system, and Agilent 2100 biological analyzer were used to ensure the quality, quantity, purity and integrity of the RNA samples. Finally, the Illumina HiSeq 2500 was used to sequence the samples.

#### 2.5.2 Expression differential gene analysis

DESeq was used to perform differential gene expression analysis, and differentially expressed genes were evaluated for expression difference multiplicity |log2FoldChange| > 1.5 and significance *p* < 0.05. In each comparison group, the number of upregulated differential genes and downregulated differential genes was counted.

#### 2.5.3 Functional enrichment analysis

Two-way cluster analysis was performed by using the R language Pheatmap software package on the concatenated sets of differential genes and samples from all comparison groups, clustering according to the expression levels of the same gene in different samples and the expression patterns of different genes in the same sample, using the Euclidean method to calculate distances and the hierarchical clustering longest distance method (Complete Linkage) for cluster analysis. In addition, GO enrichment analysis was performed using top GO and KEGG enrichment analysis was performed using the cluster profiler in this experiment.

### 2.6 Integration analysis of transcriptomic and network analysis

In this section, the back-regulated differential genes obtained from transcriptomics sequencing were integrated and analyzed with the major targets of YQW for the treatment of T2DM, taking the shared targets or shared pathways to understand the mechanism of action of YQW intervention in T2DM rats.

## 3 Results

### 3.1 Therapeutic effect of YQW in STZ-induced T2DM rats

**FIGURE 1:**
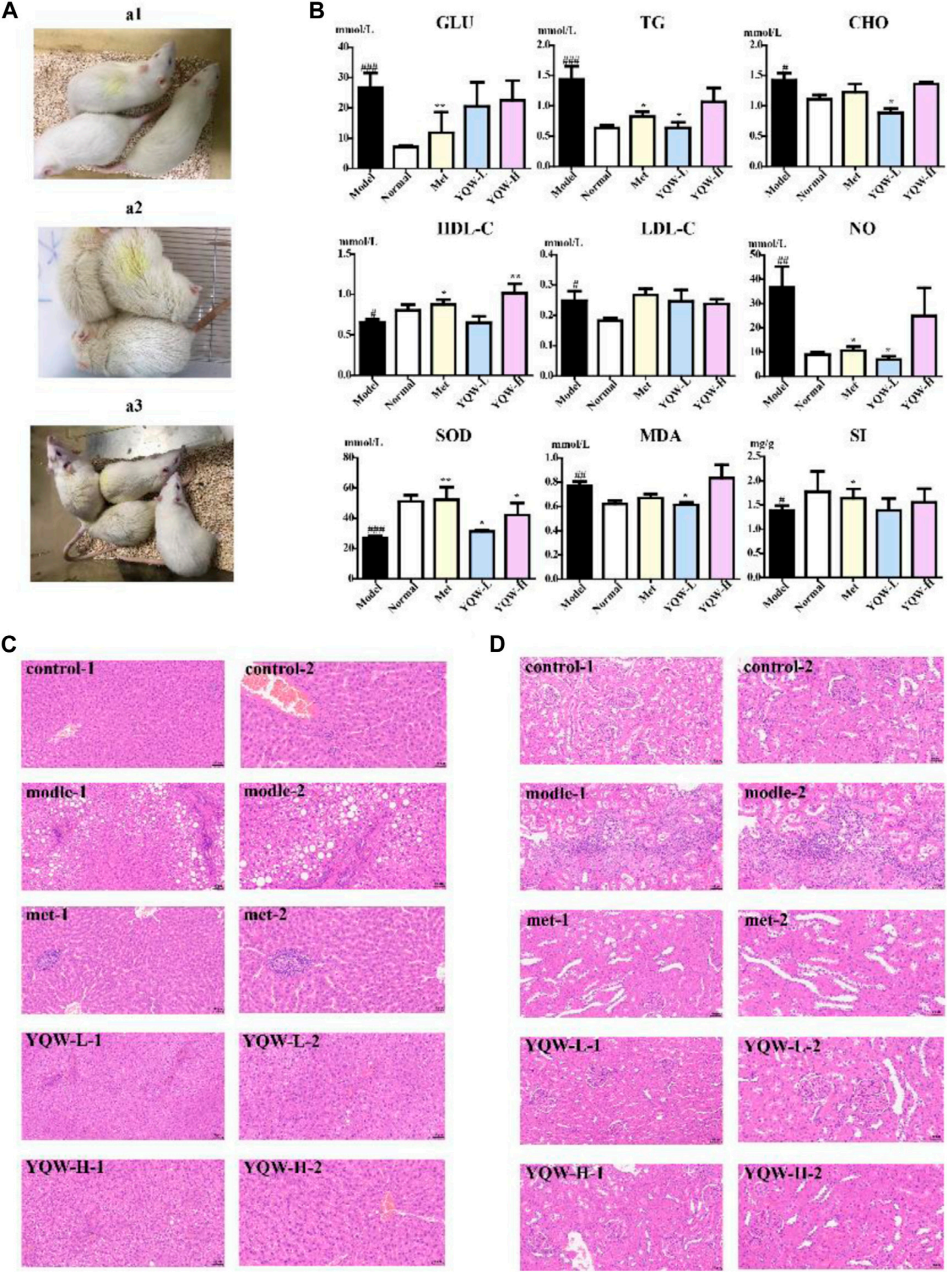
Therapeutic effect of YQW in STZ-induced T2DM rats. **(A)** The condition of each group: a1: the control group; a2: the model group; a3: the administration group. **(B)** The biochemical indexes of rats in each group. **(C)** HE staining of rat livers in each group:1:x20; 2:x200. **(D)** HE staining of rat kidney in each group:1:x20; 2:x200. Values are presented as the mean ± SD. ^###^ *p* < 0.001, ^##^ *p* < 0.01, ^#^ *p* < 0.05 versus the control group. ***p < 0.001, **p < 0.01, *p < 0.05 versus the model group.

Rats in the control group had normal drinking, diet and excretion, clean fur, good mental state and active behavior. On the contrary, the rats in the model group had obvious excessive drinking, excessive urination, thin body, rough and wet fur, poor mental state and wet bedding, which needed to be changed every day. As shown in Figure 1A, after 4 weeks of intervention, YQW group can obviously improve the mental state of rats, with smooth hair, reduced urine output and clean bedding. In addition, As shown in Figure 1B, compared with the control group, the blood glucose (GLU) of rats in the model group increased (^###^ *p* < 0.001), indicating that the modelling was successful. The GLU of rats in the Met group decreased (**p < 0.01), indicating a better hypoglycaemic effect, while the GLU in the YQW-L and YQW-H groups showed a trend of decrease compared with the model group, but there was no significant difference compared with the model group. In addition, the YQW-L group reduced TG and CHO levels in T2DM rats (*p < 0.05) and was more effective than the Met group. Besides, compared with the model group, NO level was reduced in the Met and YQW-L groups (*p < 0.05), but there was no statistical significance in the YQW-H group. Compared with the model group, SOD level was increased in the Met group (**p < 0.01). Compared with the model group, the YQW-L group (*p < 0.05) showed a significant decrease in MDA level, while the Met group and YQW-H groups showed no statistically significant decrease. These results showed that the YQW-L group was able to regulate the increase in serum NO and MDA levels and the decrease in SOD levels in rats, and enhance the antioxidant capacity of rats. Finally, compared with the model group, the Met groups showed an increase in spleen index (*p < 0.05), and the YQW-L and YQW-H groups showed a tendency to increase, although there was no statistical difference, the original data could be found in Supplementary Tables Ⅰ–Ⅳ. Then, the liver and kidney tissues of rats in each group were stained with HE. As shown in Figure 1C, in the control group, the liver lobules were clearly structured, the hepatic cords were neatly arranged, the hepatocytes were rich in cytoplasm, the morphology was normal, the hepatic sinusoids were not significantly dilated or extruded, and there was no obvious inflammation. On the contrary, in the model group, there was a large amount of hepatocyte steatosis around the confluent area, round vacuoles of different sizes were visible in the cytoplasm, and there were many focal infiltrations of lymphocytes around the confluent area. In the Met group, mild fatty degeneration of hepatocytes and small round vacuoles in the cytoplasm of the liver were seen locally, and focal infiltration of lymphocytes was seen in many places accompanying with reduced inflammation. Compared with the model group, mild fatty degeneration of hepatocytes around the confluent area and small round vacuoles in the cytoplasm of the liver were seen in the YQW-L and YQW-H group accompanying with no obvious inflammation. As for the kidney tissue, As shown in Figure 1D, the control group showed uniform staining, clear demarcation of the renal cortical medulla, normal glomerular morphology and structure, no obvious inflammation, a large number of tubular lumen with shed epithelial cells. However, the model group showed localized tubular atrophy with more lymphocytic infiltration, and more tubular lumen with shed epithelial cells. The Met group showed more tubular dilatation and a small amount of tubular epithelial cytoplasmic vacuolation, with no obvious inflammation. As for the YQW-H and YQW-L groups, the kidney tissue showed uniform staining, clear demarcation of the renal cortical medulla, normal glomerular morphology and structure, no obvious inflammation, and little tubular epithelial cytoplasmic vacuolation compared with the model group.

### 3.2 Qualitative analysis of the chemical composition of YQW

**FIGURE 2:**
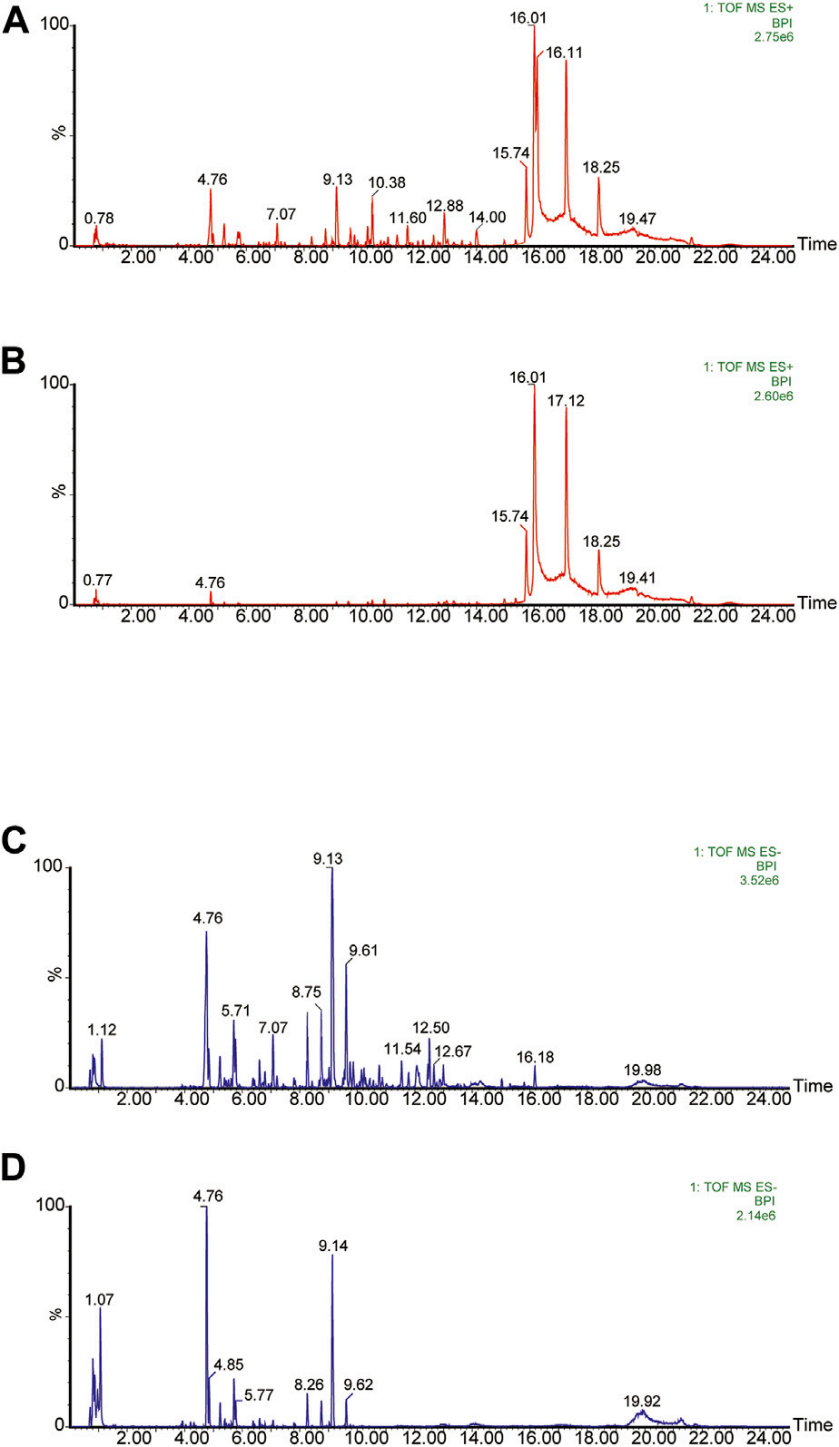
The basic peak ion current diagram of chemical composition of YQW. **(A)** Positive ion mode of 75% alcohol extract of YQW. **(B)** Positive ion mode of water extract of Yuquan pill. **(C)** Negative ion mode of 75% alcohol extract of YQW. **(D)** Negative ion mode of water extract of YQW.

75% methanolic and aqueous extracts of YQW were analysed respectively in positive and negative ion mode and the basal peak ion flow diagrams are shown in Figure 2. The mass spectral data of them were collected and automatically matched and identified by the UNIFI data processing system to obtain the retention time, precise molecular weight, error and high-energy fragmentation ion peak information of each peak.

A preliminary characterization of the 116 components in YQW was carried out by combining database comparison and literature reports. Among them of 20 components were derived from Gegen, 4 components from Dihuang, 3 components from Tianhuafen, 10 components from Maidong, 21 components from Wuweizi, and 58 components from Gancao. The main components include 59 flavonoids, 10 lignans, 9 triterpenes, 6 phenylpropanoids, 5 terpenoid, 5 steroids and 4 fatty acid, etc. The results are shown in Supplementary Material.

### 3.3 Qualitative analysis of *in vivo* blood entry prototype components of YQW

**FIGURE 3:**
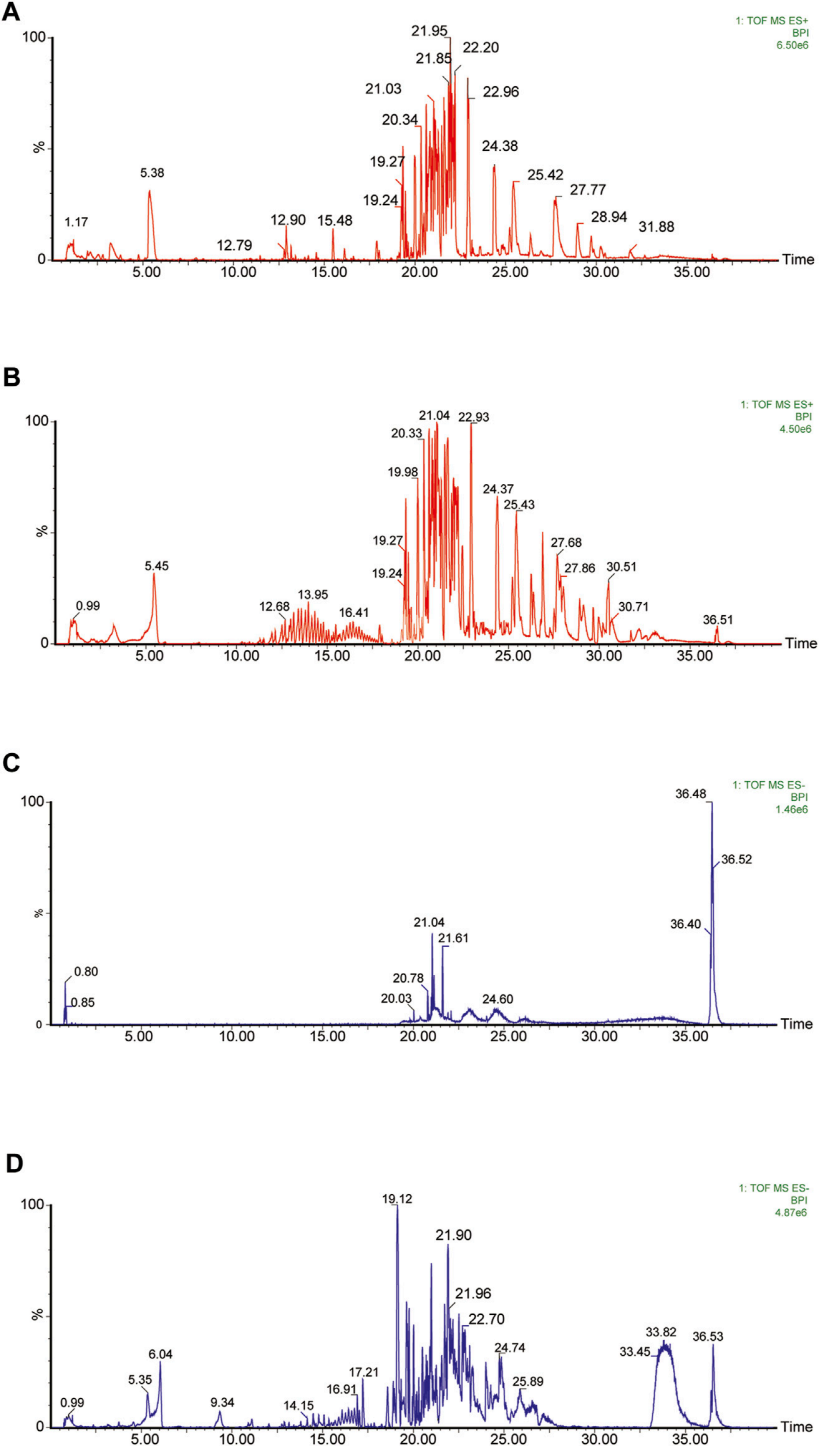
The basic peak ion current diagram of prototype components of YQW in different doses. **(A)** Positive ion mode of medicated serum in YQW-H group. **(B)** Positive ion mode of medicated serum in YQW-L group. **(C)** Negative ion mode of medicated serum in YQW-H group. **(D)** Negative ion mode of medicated serum in YQW-L group.

Blank serum and YQW-containing serum were analysed in positive and negative ion mode according to the analytical conditions established in the UPLC-Q-TOF/MS technique, and the base-peak ion flow diagrams and the table of incoming blood prototype components are shown in Table 1 and Figure 3 respectively.

**TABLE 1:** Table of prototype components identification of YQW in blood.

NO.	RT (min)	Formula	Theoretical *m/z*	Observed *m/z*	Mass error (ppm)	Adducts	MS/MS	Component	Structure	Source
1	1.49	C_10_H_13_N_5_O_4_	268.1046	268.1034	4.5	[M+H]^+^	203.0520 [M+H-NH_3_-H_2_O-CH_3_O]^+^	adeninenucleoside	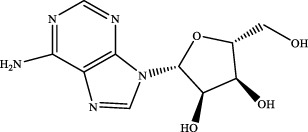	Maidong
							136.0606 [M+H-C_5_H_9_O_4_]^+^			
2	2.28	C_12_H_16_O_3_	247.0737	247.0755	−7.3	[M+K]^+^	NA	elemicin	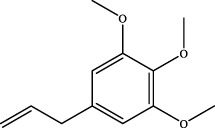	Wuweizi
3	2.88	C_21_H_16_O_6_	365.1025	365.1043	−4.9	[M+H]^+^	305.0849 [M+H-CH_3_-CHO_2_]^+^	gancaonin f	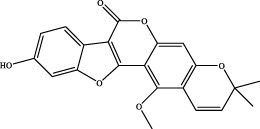	Gancao
4	9.74	C_21_H_20_O_9_	417.1186	417.1172	3.4	[M+H]^+^	161.0174 [M+H-C_12_H_12_O_3_]^+^	puerarin	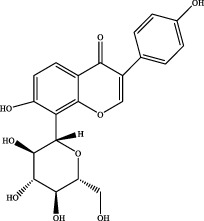	Gegen
							297.0752 [M+H-C_4_H_8_O_4_]^+^			
							267.0648 [M+H-C_5_H_10_O_4_-H_2_O]^+^			
5	10.96	C_15_H_10_O_4_	255.0657	255.0646	4.3	[M+H]^+^	NA	daidzein	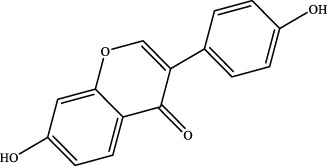	Gegen
6	12.35	C_21_H_20_O_10_	433.1135	433.1121	3.2	[M+H]^+^	365.1037 [M+H-4H_2_O]^+^	genistein 7-glucoside	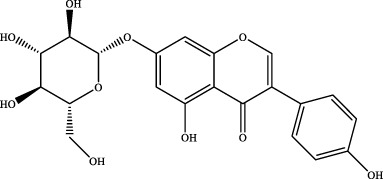	Gegen
							137.0228 [M+H-C_6_H_11_O_5_-C_8_H_5_O_2_]^+^			
7	12.54	C_15_H_12_O_4_	257.0814	257.0805	3.5	[M+H]^+^	137.0228 [M+H-C_8_H_8_O]^+^	liquiritigenin	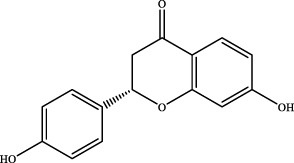	Gancao
8	14.06	C_21_H_22_O_9_	441.1162	441.1145	3.9	[M+Na]^+^	NA	liquiritin	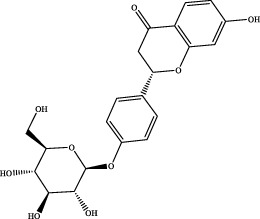	Gancao
9	14.15	C_16_H_26_O_7_	331.1757	331.1721	10.9	[M+H]^+^	167.0818 [M+H-H_2_O-C_10_H_15_O]^+^	schizonepetoside a	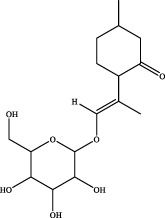	Wuweizi
10	15.74	C_15_H_12_O_4_	257.0814	257.0806	3.1	[M+H]^+^	137.0228 [M+H-C_8_H_7_O]^+^	isoliquiritigenin	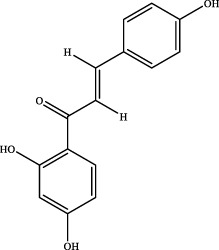	Gancao
11	19.43	C_30_H_44_O_4_	491.3137	491.3181	−9.0	[M+Na]^+^	317.2108 [M+H-C_10_H_16_O]^+^	glabrolide	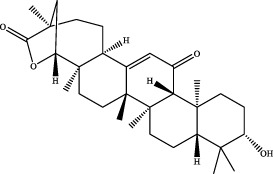	Gancao
						[M+H]^+^	217.1177 [M+H-C_16_H_25_O_2_]^+^			
12	19.76	C_23_H_28_O_6_	401.1964	401.1953	2.7	[M+H]^+^	162.0212 [M+H-C_14_H_20_O_3_]^+^	gomisin n	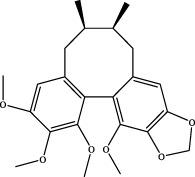	Wuweizi
13	20.24	C_22_H_24_O_7_	401.1600	401.1593	1.7	[M+H]^+^	341.1011 [M+H-C_3_H_6_O]^+^	gomisin r	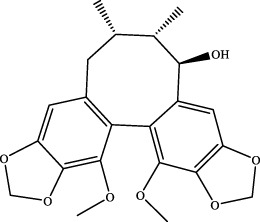	Wuweizi
14	20.59	C_30_H_44_O_5_	485.3276	485.3276	0	[M+H]^+^	119.0851 [M+H-H_2_O-CH_3_-C_20_H_24_O_4_]^+^	liquoric acid	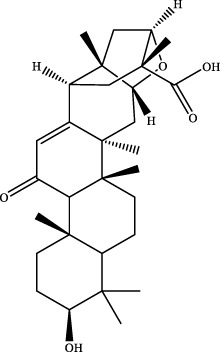	Gancao
15	20.9	C_10_H_16_O	153.1279	153.1270	5.9	[M+H]^+^	NA	citral	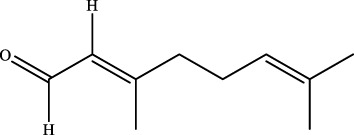	Wuweizi
16	21.09	C_30_H_46_O_4_	471.3474	471.3463	2.3	[M+H]^+^	184.1467 [M+H-C_19_H_27_O_2_]^+^	glycyrrhetinic acid	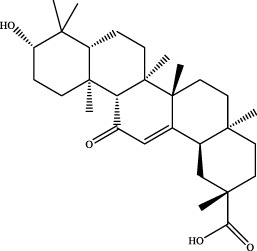	Gancao
17	19.44	C_23_H_30_O_5_	409.1991	409.1957	8.3	[M+Na]^+^	209.0477 [M+Na-C_10_H_14_-C_3_H_7_]^+^	robustadial a (Thomas, 2022)	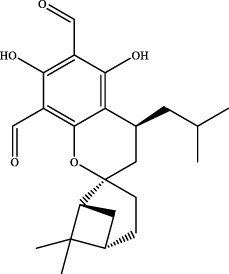	Wuweizi
18	19.45	C_22_H_22_O_10_	491.1190	491.1188	0.4	[M+HCOO]^-^	NA	3′-methoxypuerarin (Thomas, 2022)	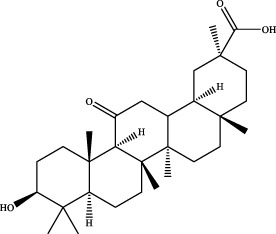	Gegen
19	20.45	C_15_H_22_O	219.1749	219.1743	2.7	[M+H]^+^	105.0694 [M+H-C_3_H_5_-C_4_H_5_O]^+^	nootkatone (Thomas, 2022)	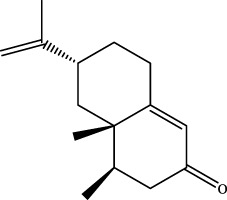	Wuweizi
20	20.98	C_28_H_48_O_2_	439.3552	439.3564	−2.7	[M+Na]^+^	303.2309 [M+H-C_8_H_17_]^+^	vitamin e (beta) (Kapoor et al., 2021)	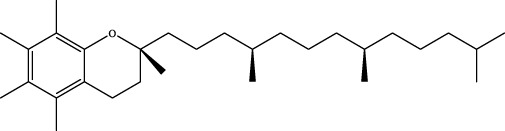	Wuweizi
						[M+H]^+^				
21	21.08	C_30_H_46_O_4_	469.3318	469.3316	0.4	[M-H]^-^	233.1538 [M-H-C_15_H_23_O_2_]^+^	18alpha-glycyrrhetinic acid (Thomas, 2022)	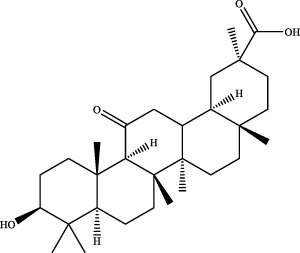	Gancao
22	36.48	C_48_H_78_O_17_	925.5161	925.5083	8.4	[M-H]^-^	581.3095 [M-H-C_2_H_2_O_3_-C_19_H_28_O]^+^	kaikasaponin iii (Thomas, 2022)	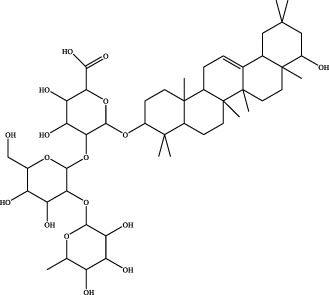	Gegen

The retention time, precise molecular weight, error and high-energy fragmentation ion peak information of each peak were obtained by comparing the ion flow maps of blank serum and YQW-H and YQW-L containing serum. UNIFI data processing system was used to automatically match and identify them. Combined with the results of the *in vitro* chemical composition characterization, a preliminary characterization of the 22 prototypical components of YQW into blood after high and low administration to T2DM rats was carried out. They were listed in Table 2. So these blood entry prototype components may be potential important substances of YQW in the intervention of T2DM rats.

**TABLE 2:** The table of blood components in YQW.

NO.	Component	Formula	Source
1	puerarin	C_21_H_20_O_9_	Gegen
2	daidzein	C_15_H_10_O_4_	Gegen
3	genistein 7-glucoside	C_21_H_20_O_10_	Gegen
4	3′-methoxypuerarin	C_22_H_22_O_10_	Gegen
5	kaikasaponin iii	C_48_H_78_O_17_	Gegen
6	adeninenucleoside	C_10_H_13_N_5_O_4_	Maidong
7	elemicin	C_12_H_16_O_3_	Wuweizi
8	schizonepetoside a	C_16_H_26_O_7_	Wuweizi
9	gomisin n	C_23_H_28_O_6_	Wuweizi
10	gomisin r	C_22_H_24_O_7_	Wuweizi
11	citral	C_10_H_16_O	Wuweizi
12	robustadial a	C_23_H_30_O_5_	Wuweizi
13	nootkatone	C_15_H_22_O	Wuweizi
14	vitamin e (beta)	C_28_H_48_O_2_	Wuweizi
15	gancaonin f	C_21_H_16_O_6_	Gancao
16	liquiritigenin	C_15_H_12_O_4_	Gancao
17	liquiritin	C_21_H_22_O_9_	Gancao
18	isoliquiritigenin	C_15_H_12_O_4_	Gancao
19	glabrolide	C_30_H_44_O_4_	Gancao
20	liquoric acid	C_30_H_44_O_5_	Gancao
21	glycyrrhetinic acid	C_30_H_46_O_4_	Gancao
22	18alpha-glycyrrhetinic acid	C_30_H_46_O_4_	Gancao

### 3.4 Retrieval results of targets, “TCM-component-target” network and functional analysis

**FIGURE 4:**
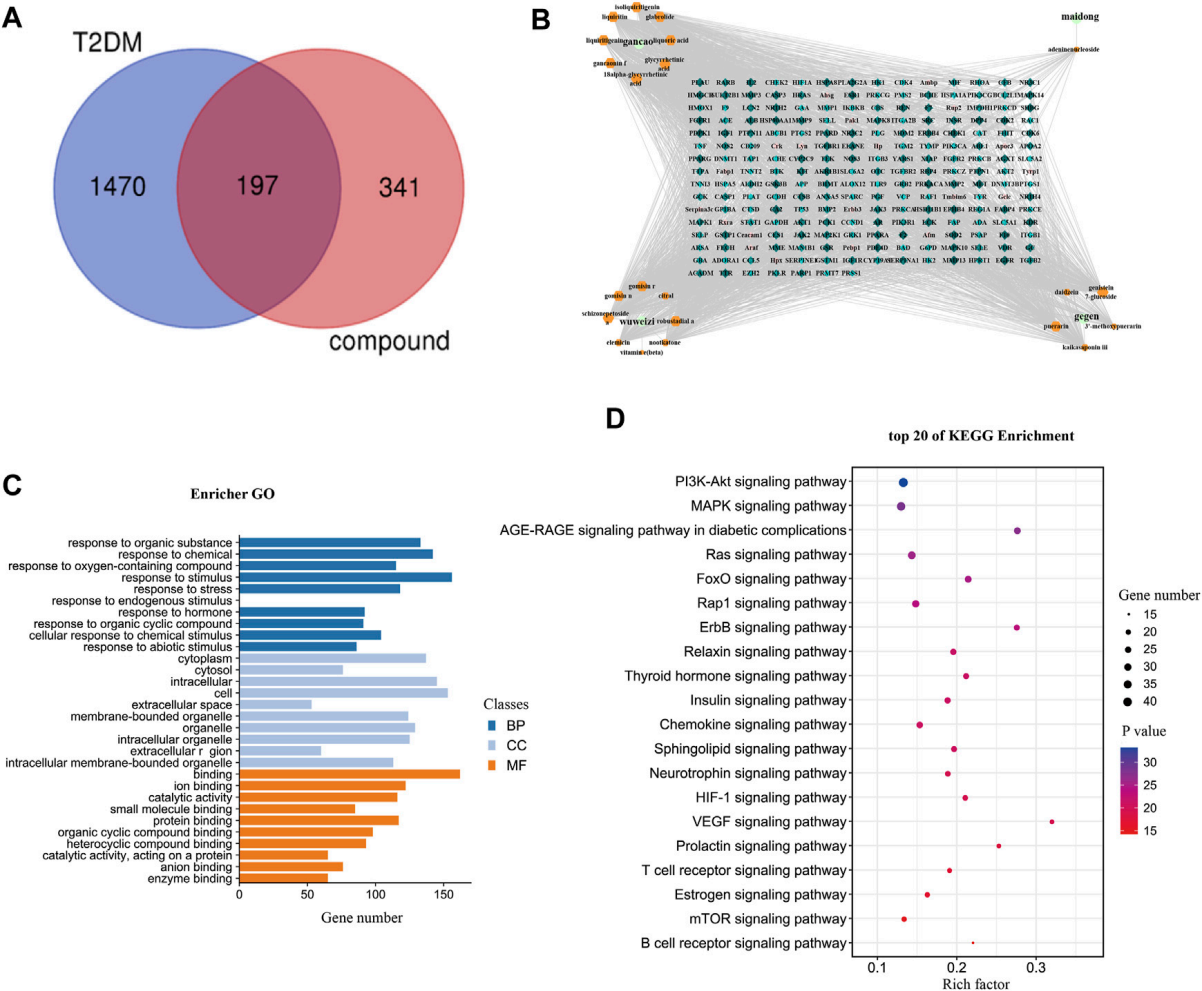
Bioinformatics analysis of potential active compound targets. **(A)** Venn diagram of chemical composition target of YQW-target of-T2DM. **(B)** Cross-mapping of “Traditional Chinese Medicine-Composition-Target” Network. The green circle in the picture represents the taste of TCM; Orange hexagon represents the chemical composition, and its size represents the Degree value; The middle diamond represents the direct target, the pink diamond represents the indirect target, and its size and color intensity represent the Degree value. It indicates the degree of interaction between target proteins, and its thickness indicates the value of Combine score. The thicker the edge, the greater the value of Combine score. **(C)** GO enrichment analysis of potential targets of YQW. **(D)** Enrichment analysis of KEGG pathway of YQW’s potential target. The abscissa is Rich Factor, and the larger the value, the greater the enrichment degree; The ordinate is the top 20 pathways with high enrichment degree; The color from red to purple indicates that the *p*-value is getting bigger and bigger, and the enrichment is becoming more and more obvious.

The 197 direct common targets were obtained by integrating 22 chemical composition targets and T2DM targets. The Venn diagram results are shown in Figure 4A. Then 197 targets were uploaded to the GeneMANIA protein interaction platform for analysis and 20 indirect targets was obtained. The inverse pharmacophore screening also revealed that all the 22 blood entry prototypes are important ingredients in YQW for the treatment of T2DM. Based on the Degree value, the top5 targets, namely, Akt1, Alb, Tp53, Casp3 and Src, were selected as the key targets of YQW for the treatment of T2DM. The “TCM-Chemistry,” “Chemistry-Direct Target” and “Direct Target-Indirect Target” property files were imported into Cytoscape V3.8.0. The network diagram of “TCM - component - direct target - indirect target” was constructed by the Merge integration function, and the results were shown in Figure 4B. The GO bioassay was visualised on the MicrolifeInfo platform, as shown in Figure 4C. The enrichment analysis of the KEGG signalling pathway based on *p*-values was carried out on the Omicshare platform to obtain bubble maps. The KEGG pathway enrichment analysis showed that of the 203 individual signalling pathways obtained, the top 20 signal transduction process were shown respectively in as shown in Figure 4D.

### 3.5 Transcriptomics

#### 3.5.1 Identification of differential genes

To further understand the multifaceted mechanism of action of YQW-L inT2DM rats, RNA sequencing analysis were performed to obtain the mRNA expression of each sample in the control, model and YQW-L groups. By comparing the differential gene expression in each group, the screening revealed that the YQW-L group could reverse the abnormally high expression of 28 genes and the abnormally low expression of 61 genes the modle group compared with the normal group. The differential gene regressions are shown in Table 3.

**TABLE 3:** The table of differential gene identification.

Gene	Up	Down	Gene	Up	Down
Pcdh18	√		Ccnf	√	
Hhex	√		LOC108348128	√	
Tmem164	√		Dnah8	√	
Gpsm2	√		Rhoh	√	
Sorbs3	√		Tnfrsf4	√	
R3hcc1l	√		LOC100912564	√	
Dsc2	√		Wfdc21	√	
Pcare	√		Nrg1	√	
AABR07012583.2	√		LOC103694879	√	
Dbp	√		Dck	√	
Wdr92	√		Cxcl14	√	
Cele2a	√		Acad10		√
Nptx2	√		Tymp		√
Epb42	√		Tgif1		√
AABR07021804.1	√		Mgat4a		√
Spata46	√		Rasgef1b		√
Mmel1	√		Ppard		√
Chka		√	Rarres1		√
Foxo1		√	Ppp1r13l		√
AABR07063279.1		√	Pkib		√
Kit		√	Ptpdc1		√
Arntl		√	Gsn		√
Thbs1		√	Bcl2l11		√
Steap3		√	Jdp2		√
Fam89a		√	Coq8a		√
Serpina5		√	Esrrg		√
Per1		√	Noct		√
Tsku		√	Nav3		√
Samd4a		√	Pax8		√
Gstt1		√	Foxp1		√
Ppargc1a		√	Slc45a3		√
Gckr		√	Col27a1		√
Pkdcc		√	Tsc22d3		√
Wfdc2		√	Fam169b		√
Sox5		√	Stard4		√
Foxo3		√	Fzd7		√
Hes1		√	Ppara		√
IL10		√	Sdc4		√
Pfkfb3		√	Cep85l		√
Zbtb16		√	Rgs9bp		√
Gpatch4		√	Veph1		√
AABR07040840.1		√	Tfap4		√
Baiap2l1		√	Cyp4f39		√
Fabp4		√	Lgsn		√
Lpin1		√			

**FIGURE 5:**
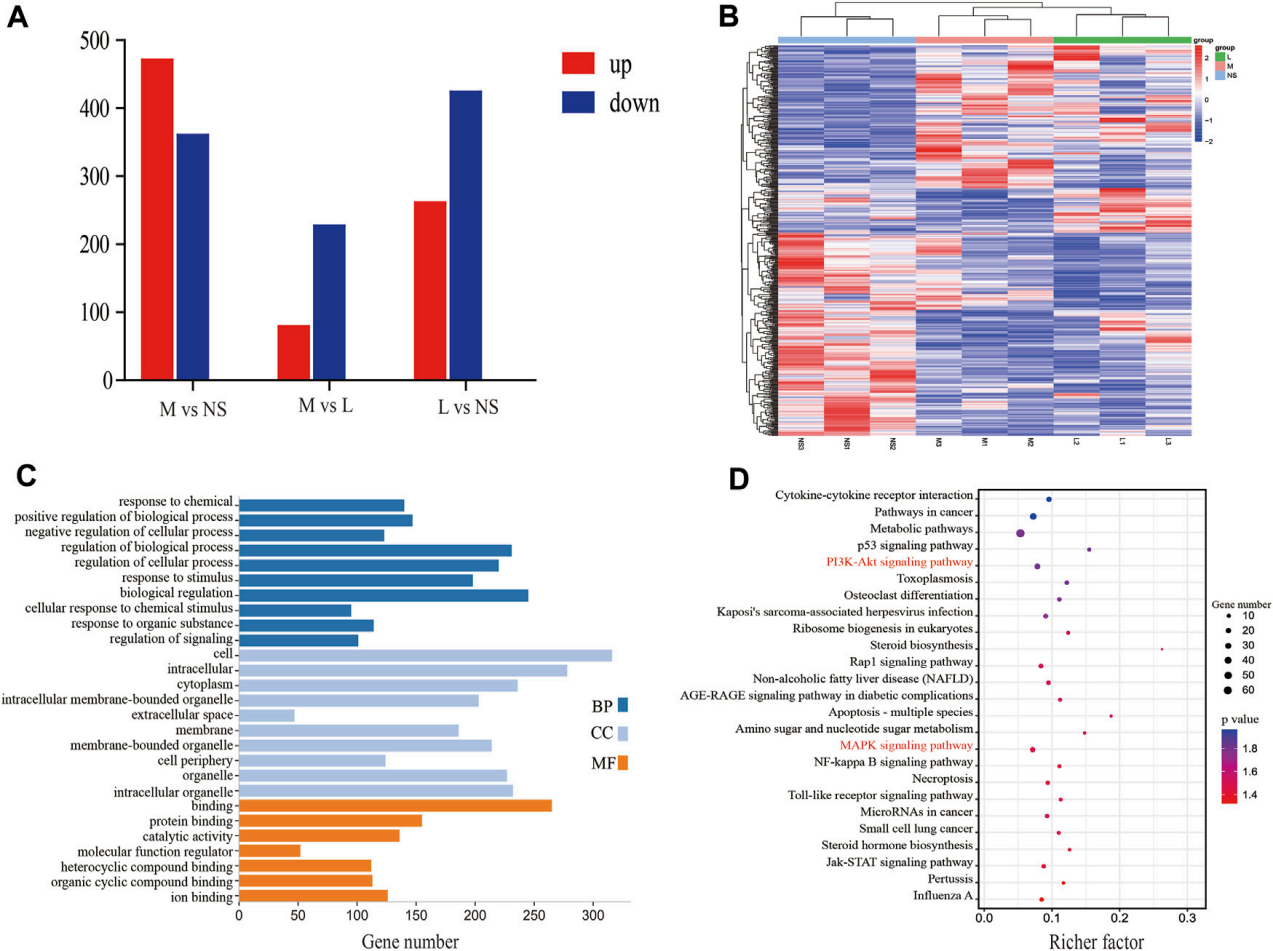
Bioinformatics analysis of genes related to treatment of T2DM with YQW. **(A)** Histogram of differential gene expression in each group. Red indicates upward adjustment and blue indicates downward adjustment; M represents the model group, NS represents the control group, and L represents the low-dose YQW administration group **(B)** Heat map of cluster analysis of different genes in each group. Blue represents the control group, red represents the model group, and green represents the low-dose YQW administration group. **(C)** GO analysis chart of differential genes in disease state. **(D)** Differential KEGG enrichment map.

Additional differential gene Venn diagrams and cluster analysis heat maps were used to represent the number of differential genes between the comparison groups, the overlap between the comparison groups and the correlation of expression between 9 individual samples in the three groups. The differential gene expression histogram and the cluster analysis heat map for each group were shown in Figures 5A, B. In comparison to the control group, 471 genes in the model group were highly expressed and 360 genes were low expressed using bioinformatics analysis technologies. In comparison to the YQW group, the model group had 79 highly expressed genes and 227 low expressed genes. In the normal group, 261 genes were highly expressed and 424 genes were low expressed as compared to the YQW group. At the same time, the cluster analysis thermogram revealed that following injection, some genes in T2DM rats’ liver tissue tended to call back to the control group, compared to the control group.

#### 3.5.2 Results of GO, KEGG pathway analysis

In this section, the expression of differential genes in the model group compared to the normal group (i.e., in the disease state) and the differential genes back-regulated in the YQW-L group compared to the normal group were analysed respectively for GO Term enrichment. In addition, KEGG processes are mainly divided into metabolic processes, environmental information processing processes, disease processes, tissue system processes and cellular processes. The differential genes and the KEGG enrichment analysis of the retraced genes in the model group compared to the normal group (i.e., in the disease state) were also shown in Figures 5C, D. In comparison to the control group, the Toll 20 pathways in the model group primarily include Rap1 signaling, AGE-RAGE signaling pathway in diabetic complications, amino acid and nucleotide sugar metabolism, MAPK signaling pathway, nuclear factor-B signaling pathway, toll-like receptor signaling pathway, PI3K-AKT signaling pathway, and so on.

#### 3.5.3 Results of integration analysis

**FIGURE 6:**
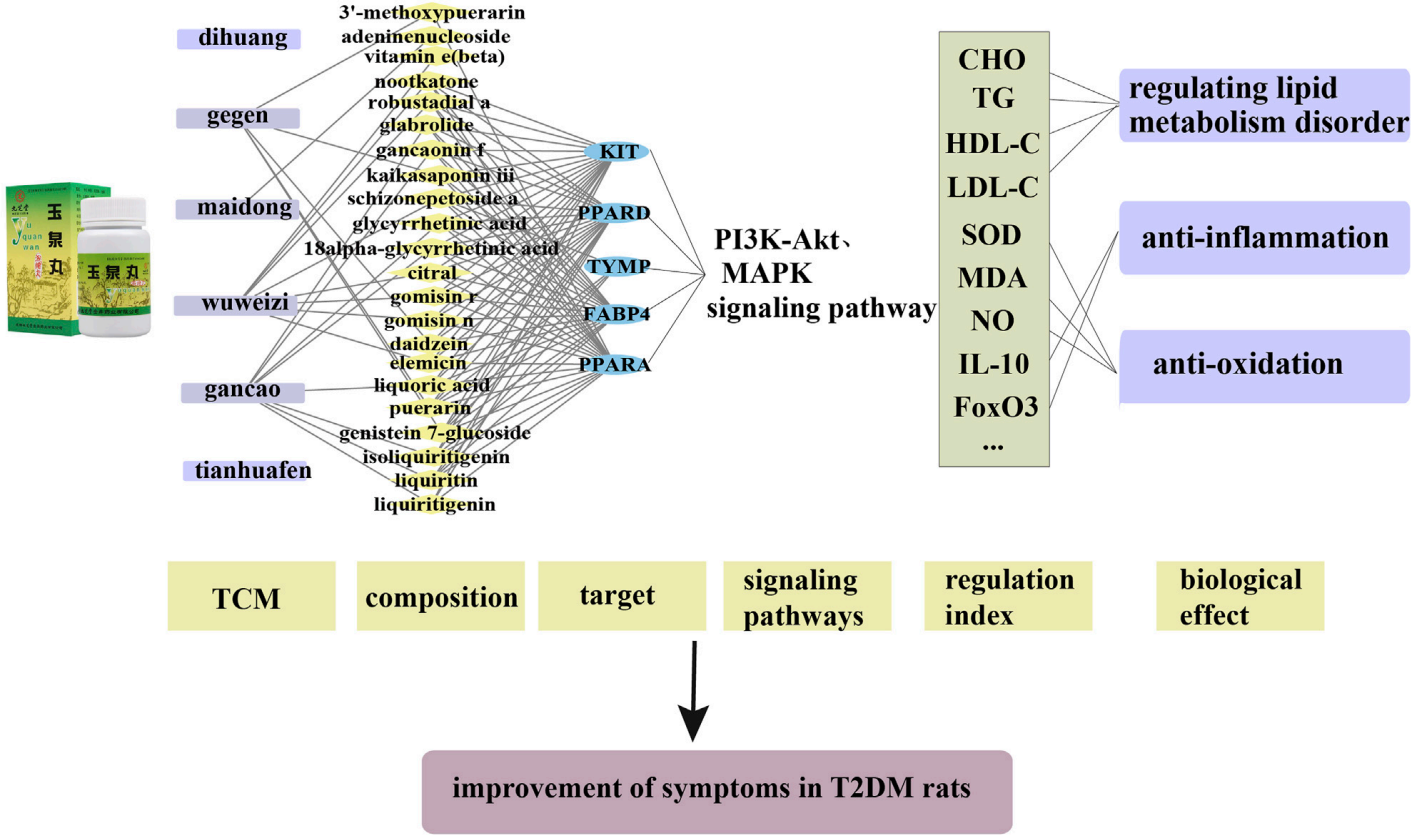
Full-text summary diagram.

Five shared targets of Kit, Ppard, Ppara, Fabp4 and Tymp were obtained by crossing the targets of pharmacodynamic components and disease predicted by network analysis with the differential targets obtained by transcriptome sequencing. And the shared passway were PI3K-Akt and MAPK signal pathway. As shown in Figure 6, this shared target and pathway could be a potential key target and pathway for YQW in the treatment of T2DM.

## 4 Discussion

### 4.1 Analysis of low hypoglycemic effect of TCM

Diabetes is a chronic disease with various consequences, and its prevalence is quickly increasing over the world, putting people’s health at risk (Nolan et al., 2011). The T2DM rats model was effectively duplicated in this study by giving them a high-fat diet for 5 weeks and injecting STZ intraperitoneally once (Sun et al., 2019). The modeling rate was 85%, and the model rats had random blood glucose of ≥16.7 mmol/L. The increase and decrease of blood glucose values in the clinical diagnosis and treatment of diabetes can be utilized as a key criterion for determining the severity and control of diabetes, so blood glucose lowering is the most critical loop in diabetes treatment (Mian et al., 2019). Our findings show that the blood glucose value of Met group decreased significantly after 4-week drug administration treatment of T2DM rats in each group. However, compared with the model group, the blood glucose value of the YQW group, whether the high-dose group or the low-dose group, showed a downward trend, but there was no significant difference. According to our literature survey, YQW is frequently used in conjunction with other TCM or western drugs in the clinical treatment of diabetes. The administration takes a little longer, about 8 weeks on average. As a result, it is still unclear if it can dramatically lower blood sugar levels in a short period of time or on its own, and more research is needed. Furthermore, findings from studies such as the United States diabetes prospective show that reducing blood sugar alone does not prevent all problems from occurring, nor does it allow all complications that have already happened to be properly treated. TCM has a high reputation for preventing and treating diabetes and its chronic consequences, although TCM has less glucose-lowering efficacy compared to western treatment (Liu et al., 2019; Wang et al., 2021a). Clinical studies have found that combining Chinese and Western medicine can shorten the time required for blood glucose to return to normal, reduce the use of Western medicine, reduce the adverse effects of Western medicine and improve symptoms such as excessive drinking, excessive urination and weight loss. Unlike single administration of Western medicine, combined TCM can take advantage of TCM’s multi-target and multi-linking effects, allowing for non-hypoglycemic diabetic treatment (Lian et al., 2015). Although the YQW group did not achieve a significant glucose reducing impact in a short period of time, it can play a role in diabetes treatment by controlling lipid metabolism abnormalities and oxidative stress levels, among other things, just like the results in our experiments.

### 4.2 Analysis of potential pharmacological substances of YQW

In addition, we created a YQW composition database with 495 components in order to elucidate the effective substances of YQW. The blood components of high and low dose YQW samples were examined based on the *in vitro* chemical properties of YQW. The precise mass number, retention period, and lysis rules were used to identify 22 chemical compounds in the blood prototype components. Flavonoids, lignans, and triterpenoids may be viable therapeutic chemicals for regulating lipid metabolism and antioxidation, such as puerarin (Zhu et al., 2010; Guo et al., 2019), gomisin n (Yun et al., 2017; Takanche et al., 2020) and glycyrrhetinic acid (Kalaiarasi and Pugalendi, 2009), according to prototype components discovered in blood research. In this section, UPLC-Q-TOF/MS technology was combined with the UNIFI data matching analysis platform to establish a rapid, sensitive, accurate, and selective blood component identification method of YQW (Zhang et al., 2021; Zhou et al., 2021), which provided reliable data and information for the follow-up network analysis to investigate the effective substances and mechanism of YQW in the treatment of T2DM.

### 4.3 Interpretation of biological significances

The integrity and systematicness of network analysis, as well as the interactions between medications and drugs and targets, tend to be congruent with TCM’s basic characteristics, which is in keeping with Chinese medicine’s understanding of disease nature (Wang et al., 2021b; Yang et al., 2022a). Therefore, applying network analysis technology to the study of the material basis and mechanism of TCM compounds can not only meet the urgent need for systematic research in TCM, but also reflect a new trend in biomedical systematic research in the era of big data (Li et al., 2022b). Previous research has shown that candidate compounds found in the serum of rats treated with TCM can be identified as active chemicals in network pharmacological analysis (Shao et al., 2022). The PI3K-Akt, MAPK, and FoXO signal pathway may be implicated in the therapeutic mechanism of YQW in treating T2DM, according to a comprehensive analysis of 538 targets and 1,667 genes associated to T2DM from 22 blood components. High-throughput mRNA sequencing technology has been used to reveal molecular mechanisms and predict biomarkers in complicated diseases including diabetes and cancer in recent years (Li et al., 2022c; Reuter et al., 2015). The YQW group can reverse the abnormal expression of 89 genes in the model group, according to our findings. These distinct genes regulate glucose and lipid metabolism, as well as anti-inflammation and anti-oxidation, mostly through the PI3K-Akt and MAPK signaling pathways, and are linked to the formation and progression of T2DM.

Furthermore, studies have revealed that islet cells produce insulin after eating. Insulin first attaches to the appropriate receptors on the cell surface, activates IRS, and transmits the insulin signal from outside the cell to the cell, and then phosphorylates FI3Kto activate Akt. The PI3K-Akt signal pathway plays a major role in lipid metabolism and glucose homeostasis by modulating growth factor signals, and it is the primary channel for insulin signal transduction. Diabetes is caused by an abnormal PI3K-Akt signal pathway. It contains important proteins that help insulin regulate cell metabolism. Insulin activates PTK and phosphorylates tyrosine residues in IRS-2, allowing p-IRS-2 to bind to PI3K and catalyze the conversion of PIP2 to PIP3. PIP3, as a second messenger, activates Akt, which subsequently controls a number of downstream proteins, including FoxO1, to stimulate glycogen production (Benchoula et al., 2021; Liu et al., 2022; Priyanka et al., 2022; Wang et al., 2022). GLP-1 can induce islet cell proliferation and suppress cell apoptosis, increase islet cell number, improve islet cell function, and stimulate insulin secretion by modulating the MAPK signaling system. The Glp-1-mediated MAPK pathway is one of the diabetes research hotspots because it plays a vital role in islet cell repair. ERK is an essential subtype of the MAPK family that is activated by phosphorylation after being induced by glucose and plays a role in islet cell proliferation and differentiation (Brown et al., 2021; El-Sayed et al., 2021). In recent years, several effector organs have been involved in research on the mechanism of anti-diabetes based on the PI3K/Akt and MAPK signaling pathways (Zhang et al., 2020). This work focused on the transcriptome of rat liver tissue and used multi-tissue and verification studies to better clarify the mechanism of YQW in the treatment of T2DM.

### 4.4 Deficiencies and limitations

*In vitro* and *in vivo*, no reference material was employed to verify YQW’s component identification, numerous active components or components with isomerism were not found, and the main active components were not quantitatively examined. As a result, in the prediction of network analysis, 22 prototype components were treated identically, which differs from the theory and characteristics of traditional Chinese medicine compound in clinical illness therapy. Pueraria lobata, Glycyrrhiza uralensis Fisch., Schisandra chinensis, and Ophiopogon japonicus were among the 22 prototyped components. In Rehmannia glutinosa and Trichosanthis Radix, no significant active components were discovered. On the one hand, this could be due to the fact that Rehmannia glutinosa is primarily a polysaccharide component, but Trichosanthis Radix is primarily composed of protein components, which do not exist as prototyped components once they enter the bloodstream. It could, on the other hand, be related to a lack of component identification. In addition to the prototype components in blood, the metabolites in YQW’s drug-containing serum must be identified further, allowing for a more thorough identification of beneficial chemicals.

Furthermore, only the expected findings from network analysis and transcriptomics results are merged and studied in the investigation of action mechanism, and five essential targets and linked important pathways require more in-depth analysis and experimental verification.

## 5 Conclusion

In summary, YQW groups can reverse the abnormally low or abnormally high expression of some genes. The active ingredients in YQW, such as puerarin, daidzein and glycyrrhetinic acid, may further activate the signalling pathways of PI3K/Akt, FoxO and AMPK by regulating the important proteins FoxO3, IL10, Ppargc1a and FoxO1, thereby reducing the level of inflammation, regulating lipid metabolism and protecting liver and kidney tissues. Five common targets, namely, Kit, Ppard, Ppara, Fabp4 and Tymp, were obtained by analyzing the targets screened with network pharmacology and the differential genes sequenced with transcriptomics, all of which were reversed after YQW administration (*p* < 0.05). This result suggests that the active ingredient in YQW may improve the lipid metabolism disorder, oxidative stress and inflammation by regulateing these 5 key genes in T2DM rats. This study looks into the potential pharmacodynamic substances and mechanism of YQW in the treatment of T2DM, but how to explain the pharmacodynamic substance basis and mechanism of YQW in the treatment of T2DM in a systematic and comprehensive manner still requires a lot of detailed research.

## Figures and Tables

**Table:** 

BP	Biological process
CHO	Cholesterol
CC	Cellular component
CTD	the Comparative toxicogenomics database
DM	Diabetes mellitus
FoXO	Forkhead box transcription factor O
GLU	Glucose
GO	Gene ontology
GLP-1	glucagon-like peptide-1
HDL-C	High density liptein cholesterol
HE	Hematoxylin-eosin
KEGG, IR	Insulin resistance; Kyoto encyclopedia of genes and genomes
LDL-C	Low density liptein cholesterol
MDA	Malondialdehyde
MAPK	Mitogen-activated protein kinase
MF	Molecular function
Met	Metformin
NO	Nitric oxide
PPI	Protein protein interaction
PKB	Protein kinase B
PI3K	Phosphatidylinositol 3-kinase
PIP2	Phosphatidylinosital biphosphate
PIP3	Phosphatidylinosital triphosphate
STZ	Streptozotocin
SOD	Superoxide dismutase
SI	Spleen index
T2DM	Type 2 diabetes mellitus
TC	Total cholesterol
TG	Triglyceride
TTD	Therapeutic target database
UPLC-Q-TOF/MS	Ultra performance liquid chromatography-quardrupole time-of-flight mass spectrometry
YQW	Yuquan pill

## Data Availability

The data presented in the study are deposited in the NCBI repository, accession number: SRP471298:PRJNA1039142.
