# Supplementary material for: Elucidating the pharmacodynamic mechanisms of Yuquan pill in T2DM rats through comprehensive multi-omics analyses
Source: Front Pharmacol. 2023 Nov 17;14:1282077. doi: 10.3389/fphar.2023.1282077 (PMC10691276; doi:10.3389/fphar.2023.1282077)
Supplement: Supplementary file 1 [file Table1.docx]

Table Ⅰ Effect of YQW for 4weeks on GLU in T2DM rats（$\bar{x}$ ± s，*n* = 6）

| Group | GLU/mmol**^.^**L**^-1^** |
| --- | --- |
| Model | 26.79 ± 4.74^###^ |
| Control | 7.10 ± 0.53 |
| Met | 11.83 ± 6.79^**^ |
| YQW-L | 20.53 ± 7.92 |
| YQW-H | 22.53 ± 6.44 |

Note: compared with the control group, ^#^ means *P* < 0.05；^##^ means *P* < 0.01；^###^ means *P* < 0.001. compared with the model group，^*^ means *P* < 0.05；^**^ means *P* < 0.01；^***^ means *P* < 0.001

Table Ⅱ Effect of YQW for 4weeks on CHO、TG、HDL-C、LDL-C in T2DM rats

（$\bar{x}$ ± s，*n* = 6）

| Group | CHO/mmol**^.^**L**^-1^** | TG/mmol**^.^**L**^-1^** | HDL-C/mmol**^.^**L**^-1^** | LDL-C/mmol**^.^**L**^-1^** |
| --- | --- | --- | --- | --- |
| Model | 1.42 ± 0.39^#^ | 1.44 ± 0.61^###^ | 0.65 ± 0.11^#^ | 0.25 ± 0.09^#^ |
| Normal | 1.11 ± 0.21 | 0.63 ± 0.13 | 0.80 ± 0.19 | 0.18 ± 0.02 |
| Met | 1.23 ± 0.38 | 0.83 ± 0.23^*^ | 0.88 ± 0.18^*^ | 0.27 ± 0.06 |
| YQW-L | 0.88 ± 0.16^*^ | 0.64 ± 0.21^*^ | 0.65 ± 0.18 | 0.25 ± 0.03 |
| YQW-H | 1.36 ± 0.07 | 1.07 ± 0.23 | 1.01 ± 0.26^**^ | 0.24 ± 0.03 |

Note: compared with the control group, ^#^ means *P* < 0.05；^##^ means *P* < 0.01；^###^ means *P* < 0.001. compared with the model group，^*^ means *P* < 0.05；^**^ means *P* < 0.01；^***^ means *P* < 0.001

Table Ⅲ Effect of YQW for 4weeks on NO、SOD、MDA in T2DM rats

（$\bar{x}$ ± s，*n* = 6）

| Group | NO/mmol**^.^**L**^-1^** | SOD/mmol**^.^**L**^-1^** | MDA/nmol**^.^**mL**^-1^** |
| --- | --- | --- | --- |
| Model | 36.74 ±27.04^##^ | 26.89 ± 3.95^###^ | 0.77 ± 0.11^##^ |
| Normal | 8.92 ± 3.75 | 51.22 ±12.20 | 0.62 ± 0.08 |
| Met | 10.63 ± 4.72^*^ | 52.22 ±25.14^**^ | 0.67 ± 0.09 |
| YQW-L | 6.97 ± 3.06^*^ | 31.40 ± 1.34^*^ | 0.61 ± 0.04^**^ |
| YQW-H | 24.91 ±25.79 | 42.25 ± 15.73^*^ | 0.83 ± 0.24 |

Note: compared with the control group, ^#^ means *P* < 0.05；^##^ means *P* < 0.01；^###^ means *P* < 0.001. compared with the model group，^*^ means *P* < 0.05；^**^ means *P* < 0.01；^***^ means *P* < 0.001

Table Ⅳ Effect of YQW for 4weeks on SI in T2DM rats（$\bar{x}$ ± s，*n* = 6）

| Group | SI/mg**^.^** g**^-1^** |
| --- | --- |
| Model | 1.39 ± 0.09^#^ |
| Normal | 1.77 ± 0.42 |
| Met | 1.64 ± 0.19^*^ |
| YQW-L | 1.39 ± 0.24 |
| YQW-H | 1.55 ± 0.28 |

Note: compared with the control group, ^#^ means *P* < 0.05；^##^ means *P* < 0.01；^###^ means *P* < 0.001. compared with the model group，^*^ means *P* < 0.05；^**^ means *P* < 0.01；^***^ means *P* < 0.001

Table V Table of chemical composition identification of YQW in vitro

| NO. | RT  (min) | Formula | Theoretical  *m/z* | Observed  *m/z* | Mass error  (ppm) | Adducts | MS/MS | Component | Type | Source |
| --- | --- | --- | --- | --- | --- | --- | --- | --- | --- | --- |
| 1 | 0.78 | C_24_H_42_O_21_ | 705.1856 | 705.1855 | 0.1 | [M+K]^+^  [M+H]^+^ | 527.1577[M+H-C_4_H_8_O_4_-H_2_O]^+^  380.0949[M+H-C_6_H_11_O_6_-C_4_H_8_O_4_]^+^ | Isolychnose | lignans | Wuweizi |
| 2 | 0.8 | C_12_H_22_O_11_ | 381.0799 | 381.0797 | 0.5 | [M+K]^+^  [M+K]^+^ | 325.1128[M+H-H_2_O]^+^  163.0594[M+H-C_6_H_12_O_6_]^+^ | gentiobiose | oligosaccharide | Gegen |
| 3 | 0.8 | C_21_H_16_O_6_ | 365.1025 | 365.1051 | -7.1 | [M+H]^+^ | 234.0968[M+H-C_8_H_4_O_2_]^+^ | gancaonin f | flavonoids | Gancao |
| 4 | 0.81 | C_15_H_20_O_5_ | 303.1208 | 303.1195 | 4.3 | [M+Na]^+^  [M+H]^+^ | 234.0968[M+H-C_2_H_4_-H_2_O]^+^  127.0386[M+H-C_3_H_4_-C_9_H_11_O_2_]^+^ | psilostachyin | [sesquiterpens](http://www.baidu.com/link?url=9a8MfoS8Nu04Ad8wjs4ZppzRXzpJeI-poEqVJ5ZZmUn9q40_a-IQLxaPBA3EdqpQbEer5Kv1Ko-BiyeIqBuxf_Gd2bbcgkThY2xZaQiroo_) | Wuweizi |
| 5 | 0.86 | C_10_H_13_N_5_O_4_ | 268.1046 | 268.1036 | 3.7 | [M+H]^+^ | 242.1021[M+H-CH_2_N]^+^  200.0704[M+H-NH_3_-3H_2_O]^+^ | adeninenucleoside | glycosides | Maidong |
| 6 | 0.86 | C_20_H_20_O_5_ | 341.1389 | 341.1367 | 6.4 | [M+H]^+^ | 309.1191[M+H-CH_3_O]^+^ | licocoumarone | flavonoids | Gancao |
| 7 | 0.86 | C_19_H_18_O_5_ | 327.1232 | 327.1223 | 2.8 | [M+H]^+^ | 164.0740[M+H-C_9_H_9_O_3_]^+^ | methy-lophiopogonone b | flavonoids | Maidong |
| 8 | 0.86 | C_15_H_22_O_4_ | 289.1416 | 289.1389 | 9.3 | [M+Na]^+^  [M+H]^+^ | 164.0740[M+H-C_3_H_7_-C_2_H_3_O_2_]^+^ | rugosal | lignans | Wuweizi |
| 9 | 0.86 | C_22_H_24_O_6_ | 385.1651 | 385.1621 | 7.8 | [M+H]^+^ | 345.1389[M+H-C_3_H_6_]^+^ | schizandrin c | lignans | Wuweizi |
| 10 | 1.13 | C_6_H_8_O_7_ | 215.0168 | 215.0157 | 5.1 | [M+Na]^+^ | NA | citric acid | fatty acids | Gegen |
| 11 | 4.2 | C_27_H_30_O_14_ | 579.1714 | 579.1705 | 1.6 | [M+H]^+^ | 417.1174[M+H-C_6_H_11_O_5_]^+^  255.0648[M+H-CH_3_O-C_11_H_19_O_9_]^+^  137.0228[M+H-C_11_H_19_O_9_-C_9_H_6_O_2_]^+^ | 4',6,7-trihydroxyisoflavone-6-methylether-7-o-β-d-xylopyranosyl-(1→6)-β-d- glucopyranoside | flavonoids | Gegen |
| 12 | 4.75 | C_17_H_14_O_5_ | 321.0739 | 321.0756 | -5.3 | [M+Na]^+^ | 281.0804[M+Na-H_2_O]^+^ | afromosin | flavonoids | Gancao |
| 13 | 4.75 | C_18_H_16_O_6_ | 351.0845 | 351.0858 | -3.7 | [M+Na]^+^  [M+H]^+^ | 297.0758[M+Na-CH_2_O]^+^  398.1830[M+H-C_3_H_4_-C_4_H_4_O_2_]^+^ | ophiopogon  anone a | flavonoids | Maidong |

Table V Table of chemical composition identification of YQW in vitro

| NO. | RT  (min) | Formula | Theoretical  *m/z* | Observed  *m/z* | Mass error  (ppm) | Adducts | MS/MS | Component | Type | Source |
| --- | --- | --- | --- | --- | --- | --- | --- | --- | --- | --- |
| 14 | 4.76 | C_21_H_20_O_9_ | 417.1186 | 417.1179 | 1.7 | [M+H]^+^ | 321.0755[M+H-2H_2_O-C_2_H_4_O_2_]^+^  297.0758[M+H-C_4_H_8_O_4_]^+^  239.0700[M+H-C_6_H_12_O_6_]^+^  165.0696[M+H-C_15_H_10_O_4_]^+^ | daidzin | flavonoids | Gegen |
| 15 | 4.76 | C_19_H_16_O_6_ | 363.0845 | 363.0860 | -4.1 | [M+Na]^+^  [M+H]^+^ | 297.0758[M+H-CHO-H_2_O]^+^ | 6-aldehydo-soophipogonone b | flavonoids | Maidong |
| 16 | 4.84 | C_26_H_28_O_13_ | 549.1608 | 549.1600 | 1.5 | [M+H]^+^ | 417.1176[M+H-C_5_H_9_O_4_]^+^  297.0756[M+H-C_9_H_16_O_8_]^+^ | mirificin | flavonoids | Gegen |
| 17 | 5.17 | C_26_H_28_O_14_ | 565.1557 | 565.1547 | 1.8 | [M+H]^+^ | 427.1001[M+H-C_4_H_4_O_4_-H_2_O]^+^  379.0814[M+H-C_5_H_9_O_4_-3H_2_O]^+^ | isoschaftoside | flavonoids | Gancao |
| 18 | 5.44 | C_21_H_20_O_10_ | 433.1135 | 433.1123 | 2.8 | [M+H]^+^ | 285.0741[M+H-C_5_H_10_O_4_-H_2_O]^+^  255.0647[M+H-C_6_H_12_O_6_]^+^ | genistein 7-glucoside | flavonoids | Gegen |
| 19 | 5.52 | C_21_H_20_O_9_ | 417.1186 | 417.1175 | 2.6 | [M+H]^+^ | 399.0753[M+H-H_2_O]^+^  321.0698[M+H-C_2_H_2_O_4_-2H_2_O]^+^  351.0693[M+H-CH_2_OH-2H_2_O]^+^ | puerarin | flavonoids | Gegen |
| 20 | 5.68 | C_21_H_20_O_9_ | 417.1186 | 417.1174 | 2.9 | [M+H]^+^ | 321.0752[M+H-C_2_H_4_O_2_-2H_2_O]^+^  239.0695[M+H -C_6_H_11_O_5_-H_2_O]^+^  147.0437[M+H-C_12_H_14_O_7_]^+^ | puerarin | flavonoids | Gegen |
| 21 | 5.7 | C_21_H_22_O_9_ | 419.1342 | 419.1316 | 6.2 | [M+H]^+^ | 321.0752[M+H-C_2_H_4_O_2_-2H_2_O]^+^  257.0803[M+H-C_6_H_11_O_5_]^+^  239.0695[M+H-C_6_H_12_O_6_]^+^ | neoliquiritin | flavonoids | Gancao |
| 22 | 5.71 | C_15_H_12_O_4_ | 257.0814 | 257.0806 | 3.1 | [M+H]^+^ | 239.0695[M+H-H_2_O]^+^  137.0230[M+H-C_8_H_7_O]^+^ | isoliquiritigenin | flavonoids | Gancao |
| 23 | 5.90 | C_15_H_10_O_5_ | 271.0606 | 271.0598 | 3.0 | [M+H]^+^ | 145.0273[M+H-C_6_H_4_O_3_]^+^ | genistein | flavonoids | Gegen |
| 24 | 6.60 | C_26_H_30_O_13_ | 551.1765 | 551.1754 | 2.0 | [M+H]^+^ | 439.1327[M+H-C_5_H_9_O_4_]^+^  257.0803[M+H-C_11_H_19_O_9_]^+^  137.0230[M+H-C_19_H_23_O_10_]^+^ | liquiritin apioside | flavonoids | Gancao |
| 25 | 6.69 | C_18_H_16_O_5_ | 313.1076 | 313.1069 | 2.2 | [M+H]^+^ | 147.0435[M+H-C_8_H_5_O_3_-CH_3_]^+^  107.0488[M+H-C_11_H_9_O_4_]^+^ | ophiopogonone b | flavonoids | Maidong |
| 26 | 6.72 | C_26_H_30_O_13_ | 551.1765 | 551.1751 | 2.5 | [M+H]^+^ | 497.1409[M+H-3H_2_O]^+^  313.1071[M+H-C_3_H_6_O_3_-C_5_H_9_O_5_]^+^  257.0800[M+H-C_11_H_18_O_9_]^+^ | neolicuroside | steroidal saponins | Gancao |
| 27 | 6.79 | C_21_H_22_O_9_ | 419.1342 | 419.1329 | 3.1 | [M+H]^+^ | 255.0803[M+H-Glu]^+^  135.0802 [M+H-Glu-C_8_H_8_O]^+^ | liquiritin | flavonoids | Gancao |

Table V Table of chemical composition identification of YQW in vitro (Continued)

| NO. | RT  (min) | Formula | Theoretical  *m/z* | Observed  *m/z* | Mass error  (ppm) | Adducts | MS/MS | | Component | Type | Source |
| --- | --- | --- | --- | --- | --- | --- | --- | --- | --- | --- | --- |
| 28 | 6.81 | C_27_H_30_O_13_ | 563.1765 | 563.1756 | 1.6 | [M+H]^+^ | 483.1246[M+H-2CH_3_O-H_2_O]^+^  441.1125[M+H-C_4_H_8_O_4_]^+^  269.0803[M+H-C_11_H_17_O_9_]^+^ | | glycyroside | flavonoids | Gancao |
| 29 | 7.01 | C_22_H_22_O_9_ | 431.1342 | 431.1331 | 2.6 | [M+H]^+^ | 269.0806[M+H-C_6_H_11_O_5_]^+^ | | 8-methoxy-5-o-glucoside flavone | flavonoids | Gancao |
| 30 | 7.05 | C_16_H_14_O_5_ | 287.0919 | 287.0909 | 3.5 | [M+H]^+^ | 255.0648[M+H-CH_3_-H_2_O]^+^  199.0748[M+H-H_2_O-C_3_H_2_O_2_]^+^ | | isogosferol | terpenoids | Gancao |
| 31 | 7.07 | C_15_H_10_O_4_ | 255.0657 | 255.0651 | 2.4 | [M+H]^+^ | NA | | daidzein | flavonoids | Gegen |
| 32 | 7.81 | C_30_H_44_O_5_ | 485.3267 | 485.3258 | 1.9 | [M+H]^+^ | 467.3157[M+H-H_2_O]^+^ | | liquoric acid | terpenoids | Gancao |
| 33 | 7.83 | C_15_H_12_O_4_ | 257.0814 | 257.0803 | 4.3 | [M+H]^+^ | NA | | liquiritigenin | flavonoids | Gancao |
| 34 | 8.27 | C_30_H_44_O_4_ | 469.3318 | 469.3310 | 1.7 | [M+H]^+^ | 451.3201[M+H-H_2_O]^+^  261.1468[M+H-C_14_H_24_O]^+^ | | glabrolide | terpenoids | Gancao |
| 35 | 8.75 | C_42_H_62_O_17_ | 839.4065 | 839.4063 | 0.2 | [M+H]^+^ | 685.3536 [M+H-C_4_H_6_O_5_-H_2_O]^+^  645.3622[M+H-C_6_H_10_O_7_]^+^ | | glyyunnanpro  sapogenin d | steroidal saponins | Gancao |
| 36 | 8.94 | C_42_H_62_O_17_ | 839.4065 | 839.4061 | 0.5 | [M+H]^+^ | 487.3404[M+H-C_12_H_17_O_12_]^+^  469.3306[M+H-C_12_H_16_O_13_]^+^  451.3202[M+H-C_12_H_16_O_13_-H_2_O]^+^ | | licoricesaponine g2 | triterpenes | Gancao |
| 37 | 8.98 | C_16_H_12_O_4_ | 269.0814 | 269.0806 | 3.0 | [M+H]^+^ | NA | | formononetin | flavonoids | Gegen |
| 38 | 9.14 | C_42_H_62_O_16_ | 823.4116 | 823.4113 | 0.4 | [M+H]^+^ | 685.3233[M+H-2H_2_O-C_5_H_5_O_2_]^+^  647.3783[M+H-C_6_H_9_O_6_]^+^  453.3361[M+H-C_12_H_15_O_13_]^+^ | | licoricesaponine k2 | triterpenes | Gancao |
| 39 | 9.14 | C_24_H_48_O_2_ | 407.3291 | 407.3302 | -2.7 | [M+K]^+^ | NA | | lignocericacid | fatty acids | Tianhuafen |
| 40 | 9.14 | C_25_H_28_O_5_ | 431.1834 | 431.1823 | 2.6 | [M+Na]^+^  [M+H]^+^ | 145.1009[M+H-C_14_H_15_O_4_-H_2_O]^+^ | 3-hydroxyglabrol | | flavonoids | Gancao |
| 41 | 9.51 | C_16_H_26_O_7_ | 369.1316 | 369.1330 | -3.8 | [M+K]^+^  [M+H]^+^ | 141.0179[M+H-C_9_H_14_O-H_2_O-CH_3_O]^+^ | schizonepetoside a | | glycosides | Wuweizi |
| 42 | 9.62 | C_42_H_65_NO_16_ | 840.4382 | 840.4372 | 1.2 | [M+H]^+^ | 685.3252[M+H-C_5_H_8_O_2_-2H_2_O]^+^  647.3786[M+H-C_6_H_9_O_6_]^+^  566.2671[M+H-C_3_H_4_O_3_-C_10_H_12_O_2_]^+^ | | monoammonium glycyrrhizinate | alkaloids | Gancao |

Table V Table of chemical composition identification of YQW in vitro (Continued)

| NO. | RT  (min) | Formula | Theoretical  *m/z* | Observed  *m/z* | Mass error  (ppm) | Adducts | MS/MS | Component | Type | Source |
| --- | --- | --- | --- | --- | --- | --- | --- | --- | --- | --- |
| 43 | 9.78 | C_15_H_24_O_5_ | 323.1261 | 323.1272 | -3.4 | [M+K]^+^  [M+H]^+^ | 175.1478[M+H-C_2_H_2_O_2_-3H_2_O]^+^ | sec-hydroxyaeginetic acid | fatty acids | Dihuang |
| 44 | 9.85 | C_39_H_64_O_14_ | 779.4194 | 779.4203 | -1.2 | [M+Na]^+^  [M+H]^+^ | 671.3744[M+H-CH_2_O-3H_2_O]^+^  321.1112[M+H-CH_2_O-H_2_O-C_25_H_38_O_3_]^+^  261.1841[M+H-C_10_H_16_O_2_-C_12_H_20_O_10_]^+^ | (25r)-samogenin 3-o  glucopyranosyl(1→2)-β-d-galactopyranoside | steroidal saponins | Tianhuafen |
| 45 | 9.87 | C_42_H_64_O_16_ | 825.4273 | 825.4264 | 1.1 | [M+H]^+^ | 671.3744[M+H-C_4_H_5_O_5_-H_2_O]^+^  613.3741[M+H-C_6_H_9_O_7_-H_2_O]^+^  455.3507[M+H-C_12_H_17_O_13_]^+^ | licoricesaponine j2 | triterpenes | Gancao |
| 46 | 10.01 | C_20_H_18_O_6_ | 355.1182 | 355.1164 | 5.1 | [M+H]^+^ | 337.1062[M+H-H_2_O]^+^ | licoisoflavaone | flavonoids | Gancao |
| 47 | 10.15 | C_20_H_18_O_6_ | 355.1182 | 355.1171 | 3.1 | [M+H]^+^ | 299.0545[M+H-C_4_H_7_]^+^  229.0488[M+H-CH_3_-H_2_O-C_6_H_5_O]^+^  165.0181[M+H-C_4_H_7_-C_8_H_6_O_2_]^+^ | licoflavonol | flavonoids | Gancao |
| 48 | 10.24 | C_28_H_48_O_2_ | 439.3552 | 439.3561 | -2.0 | [M+Na]^+^ | NA | vitamin e(beta) | vitamins | Wuweizi |
| 49 | 10.27 | C_20_H_20_O_6_ | 357.1338 | 357.1326 | 3.4 | [M+H]^+^ | 285.0754[M+H-C_4_H_7_-H_2_O]^+^  147.0439[M+H-C_4_H_7_-C_7_H_4_O_4_]^+^  135.0427[M+H-C_5_H_9_-C_7_H_4_O_4_]^+^ | sigmoidin b | flavonoids | Gancao |
| 50 | 10.27 | C_16_H_12_O_6_ | 301.0712 | 301.0703 | 3.0 | [M+H]^+^ | 285.0754[M+H-H_2_O]^+^  147.0439[M+H-H_2_O-C_7_H_6_O_3_]^+^  135.0427[M+H-C_8_H_6_O_4_]^+^ | rhamnocitrin | flavonoids | Gegen |
| 51 | 10.28 | C_21_H_20_O_6_ | 369.1338 | 369.1326 | 3.3 | [M+H]^+^ | 147.0439[M+H-C_2_H_2_-C_10_H_9_O_4_]^+^ | glycyrrhisoflavanone | flavonoids | Gancao |
| 52 | 10.38 | C_19_H_20_O_6_ | 345.1338 | 345.1319 | 5.5 | [M+H]^+^ | NA | 5,7-dihydroxy-6,8-dime thyl-3-(4'-hydroxy-3' -methoxybenzyl)chroman-4-one | flavonoids | Maidong |
| 53 | 10.38 | C_24_H_32_O_7_ | 455.2046 | 455.2037 | 2.0 | [M+Na]^+^  [M+H]^+^ | 384.1926[M+H-CH_3_O-H_2_O]^+^ | schizandrin | lignans | Wuweizi |
| 54 | 10.38 | C_21_H_22_O_5_ | 355.1545 | 355.1533 | 3.4 | [M+H]^+^ | 257.0798[M+H-C_5_H_8_-CH_3_O]^+^ | 3'-methoxyglabridin | flavonoids | Gancao |
| 55 | 10.40 | C_20_H_18_O_4_ | 323.1283 | 323.1269 | 4.3 | [M+H]^+^ | 307.1320[M+H-H_2_O]^+^ | licoflavone | flavonoids | Gancao |

Table V Table of chemical composition identification of YQW in vitro (Continued)

| NO. | RT  (min) | Formula | Theoretical  *m/z* | Observed  *m/z* | Mass error  (ppm) | Adducts | MS/MS | Component | Type | Source |
| --- | --- | --- | --- | --- | --- | --- | --- | --- | --- | --- |
| 56 | 10.43 | C_42_H_62_O_15_ | 807.4167 | 807.4153 | 1.7 | [M+H]^+^ | 631.3827[M+H-C_6_H_9_O_6_]^+^  437.3410[M+H-C_12_H_18_O_13_]^+^  419.3295[M+H-C_12_H_18_O_13_-H_2_O]^+^ | icoricesaponin c2 | triterpenes | Gancao |
| 57 | 10.66 | C_21_H_22_O_5_ | 355.1545 | 355.1526 | 5.3 | [M+H]^+^ | NA | gancaonin i | phenylpropanoids | Gancao |
| 58 | 10.68 | C_22_H_24_O_7_ | 401.1600 | 401.1588 | 3.0 | [M+H]^+^ | 353.1372[M+H-CH_3_O-H_2_O]^+^ | gomisin r | lignans | Wuweizi |
| 59 | 10.76 | C_20_H_18_O_6_ | 355.1182 | 355.1173 | 2.5 | [M+H]^+^ | 340.1290[M+H-H_2_O]^+^ | isolicoflavonol | flavonoids | Gancao |
| 60 | 10.76 | C_16_H_10_O_6_ | 299.0556 | 299.0547 | 3.0 | [M+H]^+^ | NA | isotrifoliol | phenylpropanoids | Gancao |
| 61 | 10.92 | C_23_H_28_O_7_ | 439.1733 | 439.1724 | 2.0 | [M+Na]^+^  [M+H]^+^ | 399.1792[M+H-H_2_O]^+^  330.1093[M+H-C_5_H_10_O]^+^ | schisandrol b | lignans | Wuweizi |
| 62 | 11.01 | C_20_H_18_O_5_ | 339.1232 | 339.1223 | 2.7 | [M+H]^+^ | 283.0595[M+H-C_4_H_7_]^+^ | tuberosin | flavonoids | Gegen |
| 63 | 11.04 | C_21_H_18_O_6_ | 367.1182 | 367.1169 | 3.5 | [M+H]^+^ | 327.0855[M+H-C_2_H_4_-CH_3_]^+^ | isoglycyrol | flavonoids | Gancao |
| 64 | 11.05 | C_18_H_14_O_6_ | 327.0869 | 327.0854 | 4.6 | [M+H]^+^ | NA | ophiopogonone a | flavonoids | Maidong |
| 65 | 11.16 | C_21_H_22_O_5_ | 355.1545 | 355.1531 | 3.9 | [M+H]^+^ | 321.1085[M+H-CH_3_-H_2_O]^+^ | licobenzofuran | lignans | Gancao |
| 66 | 11.23 | C_20_H_18_O_5_ | 339.1232 | 339.1219 | 3.8 | [M+H]^+^ | 283.0595[M+H-C_4_H_7_]^+^ | lupiwighteone | flavonoids | Gancao |
| 67 | 11.54 | C_20_H_20_O_4_ | 325.1440 | 325.1426 | 4.3 | [M+H]^+^ | 309.1106[M+H-CH_3_]^+^  189.0903[M+H-CH_3_-C_7_H_6_O_2_]^+^ | phaseollinisoflavan | flavonoids | Gancao |
| 68 | 11.70 | C_23_H_28_O_6_ | 401.1964 | 401.1954 | 2.5 | [M+H]^+^ | NA | gomisin n | lignans | Wuweizi |
| 69 | 11.70 | C_30_H_34_O_8_ | 523.2332 | 523.2300 | 6.1 | [M+H]^+^ | 401.1946[M+H-C_7_H_5_O-H_2_O]^+^ | benzoylgomisin h | lignans | Wuweizi |
| 70 | 11.97 | C_31_H_36_O_9_ | 553.2438 | 553.2407 | 5.6 | [M+H]^+^ | 431.2060[M+H-C_7_H_6_O_2_]^+^ | benzoylgomisin q | lignans | Wuweizi |
| 71 | 11.97 | C_22_H_22_O_6_ | 383.1495 | 383.1481 | 3.7 | [M+H]^+^ | NA | glycyrin | phenylpropanoids | Gancao |

Table V Table of chemical composition identification of YQW in vitro (Continued)

| NO. | RT  (min) | Formula | Theoretical  *m/z* | Observed  *m/z* | Mass error  (ppm) | Adducts | MS/MS | Component | Type | Source |
| --- | --- | --- | --- | --- | --- | --- | --- | --- | --- | --- |
| 72 | 11.99 | C_25_H_26_O_4_ | 391.1909 | 391.1895 | 3.6 | [M+H]^+^ | NA | hispaglabridin b | flavonoids | Gancao |
| 73 | 12.14 | C_25_H_28_O_4_ | 393.2066 | 393.2058 | 2.0 | [M+H]^+^ | 203.0698[M+H-C_12_H_15_O-CH_3_]^+^  149.0230[M+H-C_4_H_7_-C_13_H_15_O]^+^ | (e)-1-[2,4-dihydroxy-3-(3-methyl-2-butenyl)phenyl]-3-  (2,2-dimethyl-8-hydroxy-2h-benzo-pyran-6-yl)-2-propen-1-one | flavonoids | Gancao |
| 74 | 12.46 | C_20_H_28_O_5_ | 371.1834 | 371.1848 | -3.8 | [M+Na]^+^  [M+H]^+^ | 184.0731[M+H-C_11_H_18_O]^+^ | longikaurin a | phenylpropanoids | Wuweizi |
| 75 | 12.46 | C_18_H_18_O_5_ | 315.1232 | 315.1221 | 3.5 | [M+H]^+^ | NA | ophiopogonanone b | flavonoids | Maidong |
| 76 | 12.5 | C_30_H_32_O_9_ | 537.2125 | 537.2094 | 5.8 | [M+H]^+^ | 415.1747[M+H-C_7_H_5_O_2_]^+^  340.1292[M+H-C_10_H_12_O_3_-H_2_O]^+^  313.0705[M+H-C_12_H_13_O_3_-CH_3_]^+^ | gomisin g | phenylpropanoids | Wuweizi |
| 77 | 12.51 | C_25_H_28_O_6_ | 425.1964 | 425.1958 | 1.4 | [M+H]^+^ | 340.1292[M+H-C_5_H_9_-H_2_O]^+^  313.0705[M+H-2C_4_H_7_]^+^ | gancaonin e | flavonoids | Wuweizi |
| 78 | 12.67 | C_23_H_30_O_5_ | 409.1991 | 409.2004 | -3.2 | [M+Na]^+^  [M+H]^+^ | 165.0179[M+H-C_15_H_25_O]^+^ | robustadial a | others | Wuweizi |
| 79 | 12.67 | C_21_H_20_O_5_ | 353.1389 | 353.1378 | 3.1 | [M+H]^+^ | 165.0179 [M+H-C_9_H_8_O-C_4_H_7_]^+^ | gancaonin a | flavonoids | Gancao |
| 80 | 12.89 | C_25_H_28_O_6_ | 425.1964 | 425.1956 | 1.9 | [M+H]^+^ | 191.1058[M+H-C_12_H_12_O_4_-CH_3_]^+^  135.0433[M+H-C_5_H_9_-C_12_H_11_O_4_]^+^ | glisoflavanone | flavonoids | Gancao |
| 81 | 12.89 | C_21_H_20_O_6_ | 369.1338 | 369.1325 | 3.5 | [M+H]^+^ | 191.1058[M+H-C_9_H_6_O_4_]^+^  135.0433[M+H-C_13_H_14_O_4_]^+^ | glycycoumarin | flavonoids | Gancao |
| 82 | 13.00 | C_21_H_18_O_6_ | 367.1182 | 367.1171 | 3.0 | [M+H]^+^ | 311.0548[M+H-C_4_H_7_]^+^  165.0179[M+H-C_5_H_9_-C_8_H_4_O_2_]^+^  137.0233[M+H-C_5_H_9_-C_9_H_4_O_3_]^+^ | glycyrol | flavonoids | Gancao |

Table V Table of chemical composition identification of YQW in vitro (Continued)

| NO. | RT  (min) | Formula | Theoretical  *m/z* | Observed  *m/z* | Mass error  (ppm) | Adducts | MS/MS | Component | Type | Source |
| --- | --- | --- | --- | --- | --- | --- | --- | --- | --- | --- |
| 83 | 13.00 | C_25_H_26_O_6_ | 423.1808 | 423.1799 | 2.1 | [M+H]^+^ | 367.1172[M+H-C_4_H_7_]^+^  311.0548[M+H-2C_4_H_7_]^+^  165.0179[M+H-C_4_H_7_-C_13_H_13_O_2_]^+^ | glyarallin b | flavonoids | Gancao |
| 84 | 13.46 | C_21_H_20_O_5_ | 353.1389 | 353.1380 | 2.5 | [M+H]^+^ | 267.0643[M+H-C_4_H_7_-CH_3_O]^+^  189.0907[M+H-C_9_H_6_O_3_]^+^ | lyurallin a | flavonoids | Gancao |
| 85 | 13.47 | C_15_H_22_O | 219.1749 | 219.1736 | 5.9 | [M+H]^+^ | 119.0848[M+H-C_3_H_3_O-C_3_H_5_]^+^ | nootkatone | quinonoids | Wuweizi |
| 86 | 13.50 | C_30_H_46_O_4_ | 471.3474 | 471.3466 | 1.7 | [M+H]^+^ | 189.1633[M+H-C_16_H_22_O_3_-H_2_O]^+^ | glycyrrhetinic acid | triterpenes | Gancao |
| 87 | 14.59 | C_25_H_24_O_5_ | 405.1702 | 405.1689 | 3.2 | [M+H]^+^ | 349.1066[M+H-C_4_H_7_]^+^ | puerarol | flavonoids | Gegen |
| 88 | 19.05 | C_30_H_46_O_9_ | 551.3220 | 551.3269 | -8.9 | [M+H]^+^ | 495.2650[M+H-C_4_H_7_]^+^  230.1393[M+H-H_2_O-C_15_H_24_O_6_]^+^  179.0635[M+H-C_20_H_33_O_6_]^+^ | ruvoside | terpenoids | Gancao |
| 89 | 21.48 | C_9_H_16_O_4_ | 189.1127 | 189.1126 | 0.5 | [M+H]^+^ | NA | eucommiol | others | Gegen |
| 90 | 16.02 | C_10_H_14_ | 135.1174 | 135.1164 | 7.4 | [M+H]^+^ | 234.0968[M+H-C_8_H_4_0_2_]^+^  398.1830[M+H-C_3_H_4_-C_4_H_4_0_2_]^+^ | p-cymene | alkanes | Wuweizi |
| 91 | 16.02 | C_10_H_16_ | 137.1330 | 137.1320 | 7.3 | [M+H]^+^ | 107.0851[M+H-C_2_H_4_]^+^ | 1-methyl-4-methylethenylcyclohexene | alkanes | Wuweizi |
| 92 | 16.02 | C_10_H_16_O | 153.1279 | 153.1269 | 6.5 | [M+H]^+^ | 125.0955[M+H-CH_3_]^+^ | citral | others | Wuweizi |
| 93 | 1.10 | C_12_H_16_O_3_ | 231.0997 | 231.0975 | 9.5 | [M+Na]^+^  [M+H]^+^ | 180.0855[M+H-C_2_H_4_]^+^  136.0600[M+H-CH_3_O-C_3_H_5_]^+^ | elemicin | others | Wuweizi |
| 94 | 1.11 | C_21_H_22_O_4_ | 339.1596 | 339.1574 | 6.5 | [M+H]^+^ | 162.0751[M+H-C_11_H_11_O_2_]^+^  136.0600[M+H-CH_3_-C_12_H_13_O_2_]^+^ | 4'-O-methylglabridin | phenylpropanoids | Gancao |
| 95 | 1.26 | C_16_H_16_O_3_ | 257.1178 | 257.1167 | 4.3 | [M+H]^+^ | NA | orchinol | others | Maidong |
| 96 | 36.84 | C_18_H_30_O_2_ | 279.2324 | 279.2314 | 3.6 | [M+H]^+^ | 107.0851[M+H-2CH_3_]^+^ | linolenicacid | fatty acids | Tianhuafen |
| 97 | 3.92 | C_27_H_30_O_14_ | 577.1557 | 577.1565 | -1.4 | [M-H]^-^ | 457.1133[M-H-C_4_H_8_O_4_]^+^  267.0648[M-H-C_6_H_12_O_6_-C_5_H_10_O_4_]^+^ | daidzein 4',7-diglucoside | flavonoids | Gegen |
| 98 | 4.93 | C_22_H_22_O_10_ | 445.1135 | 445.1140 | -1.1 | [M-H]^-^ | 325.0710[M-H-C_4_H_8_O_4_]^+^  282.0526[M-H-C_6_H_11_O_5_]^+^  253.0498[M-H-C_6_H_11_O_5-_CH_3_O]^+^ | 3'-methoxypuerarin | flavonoids | Gegen |

Table V Table of chemical composition identification of YQW in vitro (Continued)

| NO. | RT  (min) | Formula | Theoretical  *m/z* | Observed  *m/z* | Mass error  (ppm) | Adducts | MS/MS | Component | Type | Source |
| --- | --- | --- | --- | --- | --- | --- | --- | --- | --- | --- |
| 99 | 5.02 | C_35_H_46_O_20_ | 785.2504 | 785.2497 | 0.9 | [M-H]^-^ | 623.2176[M-H-C_9_H_7_O_3_]^+^  161.0240[M-H-C_26_H_38_O_17_]^+^  133.0294[M-H-C_27_H_40_O_18_]^+^ | purpureaside c | glycosides | Dihuang |
| 100 | 5.44 | C_21_H_20_O_10_ | 431.0978 | 431.0980 | -0.5 | [M-H]^-^ | 295.0606[M-H-C_4_H_8_O_4_-H_2_O]^+^  253.0498[M-H-C_6_H_11_O_5_-H_2_O]^+^  215.0008[M-H-C_4_H_8_O_4_-C_6_H_5_O]^+^ | genistein 8-c- glucoside | flavonoids | Gegen |
| 101 | 5.62 | C_20_H_18_O_7_ | 415.1029 | 415.1033 | -1.0 | [M+HCOO]^-^  [M-H]^-^ | 252.0419[M-H-C_5_H_10_O_2_-H_2_O]^+^  223.0398[M-H-C_6_H_4_O_2_-2H_2_O]^+^ | glycyrrhiza-flavonol a | flavonoids | Gancao |
| 102 | 5.77 | C_21_H_22_O_9_ | 417.1186 | 417.1191 | -1.2 | [M-H]^-^ | 255.0662[M-H-C_6_H_11_O_5_]^+^  135.0086[M-H-C_14_H_17_O_6_]^+^ | isoliquiritin | flavonoids | Gancao |
| 103 | 5.81 | C_29_H_36_O_15_ | 623.1976 | 623.1976 | 0 | [M-H]^-^ | 461.1655[M-H-C_9_H_7_O_4_]^+^  161.0241[M-H-C_16_H_20_O_5_-C_9_H_7_O_4_]^+^ | acteoside | glycosides | Dihuang |
| 104 | 5.90 | C_21_H_20_O_10_ | 477.1033 | 477.1036 | -0.6 | [M+HCOO]^-^  [M-H]^-^ | 269.0447[M-H-C_6_H_11_O_5_]^+^  268.0371[M-H-C_6_H_12_O_5_]^+^ | 3’-hydroxypuerarin | flavonoids | Gegen |
| 105 | 6.98 | C_31_H_40_O_15_ | 651.2289 | 651.2290 | -0.2 | [M-H]^-^ | 549.1611[M-H-C_4_H_8_O_2_-CH_3_]^+^  531.1506[M-H -C_5_H_10_O_3_]^+^  475.1821[M-H-C_10_H_9_O_3_]^+^ | isomartynoside | glycosides | Dihuang |
| 106 | 9.13 | C_42_H_62_O_16_ | 821.3960 | 821.3963 | -0.4 | [M-H]^-^ | 759.3952[M-H-CHO_2_-H_2_O]^+^  697.3492[M-H-2CHO_2_-2H_2_O]^+^  645.3634[M-H-C_6_H_8_O_6_]^+^ | glyeurysaponin | triterpenes | Gancao |
| 107 | 9.75 | C_51_H_78_O_20_ | 1009.5008 | 1009.4982 | 2.6 | [M-H]^-^ | 941.5121[M-H-CHO_2_-C_2_H_2_]^+^  821.3968[M-H-C_3_H_6_-C_6_H_12_O_4_]^+^ | licoricesaponin d3 | triterpenes | Gancao |
| 108 | 9.77 | C_42_H_62_O_16_ | 821.3960 | 821.3970 | -1.2 | [M-H]^-^ | 615.3903[M-H-CHO_2_-C_5_H_8_O_5_-H_2_O]^+^  583.3636[M-H-CHO_2_-C_6_H_9_O_7_]^+^  525.3941[M-H-2CHO_2_-C_6_H_9_O_7_-H_2_O]^+^ | glycyrrhizic acid | triterpenes | Gancao |

Table V Table of chemical composition identification of YQW in vitro (Continued)

| NO. | RT  (min) | Formula | Theoretical  *m/z* | Observed  *m/z* | Mass error  (ppm) | Adducts | MS/MS | Component | Type | Source |
| --- | --- | --- | --- | --- | --- | --- | --- | --- | --- | --- |
| 109 | 10.07 | C_48_H_78_O_17_ | 925.5161 | 925.5160 | 0.1 | [M-H]^-^ | 823.4120[M-H-C_6_H_13_O]^+^  805.4012[M-H-C_6_H_13_O-H_2_O]^+^ 803.3864[M-H-CH_2_O-C_5_H_11_-H_2_O]^+^ | kaikasaponin iii | steroidal saponins | Gegen |
| 110 | 10.42 | C_42_H_62_O_15_ | 805.4010 | 805.4018 | -1.0 | [M-H]^-^ | 737.4124[M-H-CHO_2_-C_2_H_2_]^+^  629.3694[M-H-C_6_H_9_O_6_]^+^  351.0569[M-H-C_30_H_45_O_3_]^+^ | licoricesaponine c2 | steroidal saponins | Gancao |
| 111 | 10.44 | C_20_H_18_O_6_ | 353.1025 | 353.1028 | -0.8 | [M-H]^-^ | 321.1128[M-H-2H_2_O]^+^  146.9656[M-H-C_8_H_6_O -C_5_H_10_O]^+^ | gancaonin c | flavonoids | Gancao |
| 112 | 12.02 | C_44_H_70_O_16_ | 899.4640 | 899.4654 | -1.6 | [M+HCOO]^-^  [M-H]^-^ | 721.4165[M-H-C_5_H_9_O_4_]^+^  575.3582[M-H-C_5_H_9_O_4_-C_6_H_11_O_4_]^+^  367.1169[M-H-C_3_H_5_O_2_-C_27_H_45_O_3_]^+^ | 25(s)-ruscogenin 1-o-alpha-l-rhamnopy-ranosyl-(1-2)-beta-d-xylopyranoside | terpenoids | Maidong |
| 113 | 12.14 | C_25_H_28_O_4_ | 391.1909 | 391.1911 | -0.5 | [M-H]^-^ | 203.0708[M-H-C_13_H_15_O]^+^  187.1123[M-H-C_12_H_13_O_3_]^+^  132.0574[M-H-C_12_H_13_O_3_-C_4_H_7_]^+^ | (e)-1-[2,4-dihydroxy-3-(3-methyl-2-butenyl)phenyl]-3-(4-hydroxy-3-[3-methyl-2-butenyl)phenyl]-2-propen-1-one | flavonoids | Gancao |
| 114 | 13.11 | C_21_H_22_O_4_ | 337.1440 | 337.1438 | 0.6 | [M-H]^-^ | 267.0652[M-H-C_5_H_9_]^+^  201.0909 [M-H-CH_3_-C_7_H_5_O_2_]^+^  175.0755[M-H-CH_3_-C_9_H_7_O_2_]^+^ | licochalcone a | flavonoids | Gancao |
| 115 | 13.50 | C_30_H_46_O_4_ | 469.3318 | 469.3320 | -0.4 | [M-H]^-^ | 425.3423[M-H-CHO_2_]^+^  355.2636[M-H-C_6_H_10_O_2_]^+^ | 18alpha-glycyrrhetinic acid | triterpenes | Gancao |
| 116 | 4.30 | C_12_H_14_O_8_ | 285.0610 | 285.0613 | -1.1 | [M-H]^-^ | 153.0187[M-H-C_5_H_9_O_4_]^+^ | uralenneoside | glycosides | Gancao |
